# Removal of mobile genetic elements from the genome of *Clostridioides difficile* and the implications for the organism’s biology

**DOI:** 10.3389/fmicb.2024.1416665

**Published:** 2024-06-20

**Authors:** Haitham Hussain, Amer Nubgan, César Rodríguez, Korakrit Imwattana, Daniel R. Knight, Valerija Parthala, Peter Mullany, Shan Goh

**Affiliations:** ^1^Department of Microbial Diseases, Eastman Dental Institute, University College London, London, United Kingdom; ^2^Department of Clinical, Pharmaceutical and Biological Sciences, University of Hertfordshire, Hatfield, United Kingdom; ^3^Facultad de Microbiología and Centro de Investigación en Enfermedades Tropicales (CIET), Universidad de Costa Rica, San José, Costa Rica; ^4^School of Biomedical Sciences, The University of Western Australia, Perth, WA, Australia; ^5^Department of Microbiology, Faculty of Medicine Siriraj Hospital, Mahidol University, Salaya, Thailand; ^6^Department of Microbiology, PathWest Laboratory Medicine WA, Queen Elizabeth II Medical Centre, Nedlands, WA, Australia

**Keywords:** *C. difficile*, prophage deletion, transposon deletion, CRISPR-Cas9, site-specific recombinase

## Abstract

*Clostridioides difficile* is an emerging pathogen of One Health significance. Its highly variable genome contains mobile genetic elements (MGEs) such as transposons and prophages that influence its biology. Systematic deletion of each genetic element is required to determine their precise role in *C. difficile* biology and contribution to the wider mobilome. Here, Tn*5397* (21 kb) and ϕ027 (56 kb) were deleted from *C. difficile* 630 and R20291, respectively, using allele replacement facilitated by CRISPR-Cas9. The 630 Tn*5397* deletant transferred PaLoc at the same frequency (1 × 10^−7^) as 630 harboring Tn*5397*, indicating that Tn*5397* alone did not mediate conjugative transfer of PaLoc. The R20291 ϕ027 deletant was sensitive to ϕ027 infection, and contained two unexpected features, a 2.7 kb remnant of the mutagenesis plasmid, and a putative catalase gene adjacent to the deleted prophage was also deleted. Growth kinetics of R20291 ϕ027 deletant was similar to wild type (WT) in rich medium but marginally reduced compared with WT in minimal medium. This work indicates the commonly used pMTL8000 plasmid series works well for CRISPR-Cas9-mediated gene deletion, resulting in the largest deleted locus (56.8 kb) described in *C. difficile*. Removal of MGEs was achieved by targeting conjugative/integrative regions to promote excision and permanent loss. The deletants created will be useful strains for investigating Tn*5397* or ϕ027 prophage contribution to host virulence, fitness, and physiology, and a platform for other mutagenesis studies aimed at functional gene analysis without native transposon or phage interference in *C. difficile* 630 and R20291.

## 1 Introduction

*Clostridioides difficile*, also known as *Clostridium difficile* (Lawson et al., 2016), is a Gram-positive, anaerobic, endospore-forming bacterium that causes gastrointestinal illness. It is a leading cause of antibiotic-associated diarrhea (He et al., 2013; Smits et al., 2016), and recurrent *C. difficile* infection (CDI) is difficult to treat with antibiotics alone (van Prehn et al., 2021). It is an important nosocomial and community-acquired pathogen worldwide (Magill et al., 2014; Collins et al., 2020; Finn et al., 2021; Viprey et al., 2022). Sources of infection include community spaces, environmental water and soil, animals, and the food chain (Candel-Pérez et al., 2019; Knight and Riley, 2019; Jo et al., 2022). Genetically similar strains from pigs and humans have been reported, indicating possible zoonotic or anthropogenic transmission (Knetsch et al., 2014; Moloney et al., 2021).

*Clostridioides difficile* has a highly variable genome with up to 30% being made up of mobile genetic elements (MGEs) (Sebaihia et al., 2006). These are very common in bacteria and can loosely be defined as any genetic element that can mediate its own transfer from one part of the genome to another. *C. difficile* contains a range of MGEs from the very simple, such as insertion sequences (IS) to complex integrative conjugative elements (ICE, also sometimes referred to as conjugative transposons) and integrated phage genomes called prophage (reviewed in Roberts et al., 2014). These MGEs can have a profound effect on the biology of *C. difficile.* For example, ICE often encode resistance to antibiotics; e.g., Tn*916,* and Tn*5397* (tetracycline resistance). A large ICE, 023_CTnT found in *C. difficile* clade 3 strains contains genes encoding a sortase, putative sortase substrates, lantibiotic ABC transporters and a putative siderophore biosynthetic cluster (Shaw et al., 2019). Similar genes are found throughout the gut microbiome indicating that ICE have a role in allowing organisms to adapt to their local environment and can transfer through the gut microbiome. *C. difficile* also contains integrative mobilizable elements such as Tn*4453*a/b (chloramphenicol resistance), and Tn*5398* (erythromycin resistance), which spread via conjugation, between and beyond *C. difficile* (Roberts et al., 2014). Furthermore, bioinformatic analysis of *C. difficile* ICE show that they contain different sigma factors implying that they can have a global role in gene expression in the organism (Brouwer et al., 2011). Prophage and ICE are modular MGEs and both typically enter the host bacterial genome via the activity of site-specific recombinases (Johnson and Grossman, 2015). These belong to two different families namely serine or tyrosine. The amino acid named referring to that responsible for cutting DNA at the active site (Johnson and Grossman, 2015). Comparison of ICE and phage show further relationships indicating that they can exchange modules and have an intertwined evolutionary history (Johnson and Grossman, 2015).

Lysogeny is frequently observed in *C. difficile* (Sebaihia et al., 2006; Ramirez-Vargas et al., 2018), with prophages most commonly belonging to the order Siphoviridae and Myoviridae, and most commonly identified with ϕC2 (Goh et al., 2005), ϕMMP04 (Meessen-Pinard et al., 2012), ϕCD119 (Govind et al., 2006), ϕCDHM1 (Hargreaves et al., 2014), ϕCD38-2 (Fortier and Moineau, 2007), and ϕCD27 (Mayer et al., 2008), ranging in size from 31 to 56 kb with a GC content similar to that of the *C. difficile* genome (28–30%) (Knight et al., 2015). *C. difficile* prophages can influence host toxin regulation (Goh et al., 2005; Govind et al., 2009, 2011; Sekulovic et al., 2011; Riedel et al., 2017), quorum sensing (Hargreaves et al., 2014), biofilm formation (Slater et al., 2019) and fitness including transduction (Goh et al., 2013), phage immunity (Boudry et al., 2015; Sekulovic et al., 2015; Li et al., 2020), and plasmid/prophage maintenance (Peltier et al., 2020). Some of these studies were carried out by infecting *C. difficile* with a phage of interest and examining changes to the transcriptome or selected phenotype. However due to the presence of prophages in the studied strains, it can be difficult to attribute changes solely to the infecting phage.

To prove the role of ICE and prophage in *C. difficile* biology it is necessary to make clean scarless deletions of these large genetic elements. The best understood *C. difficile* ICE is Tn*5397,* which encodes resistance to tetracycline and is capable of broad host range transfer within several Gram-positive organisms (Wang et al., 2006). Tn*5379* translocates between strains by excising and forming a circular molecule, which is then capable of conjugal transfer to a suitable recipient or reintegration into the host genome (Supplementary Figure S1). In *C. difficile* 630 this element integrates into the genome close to the region of the chromosome which encodes the major virulence factors of the organism toxins A and B, termed the PaLoc (Brouwer et al., 2013). The later element can transfer at low frequency to non-toxigenic *C. difficile* strains converting them to toxin producers. The PaLoc does not have any genes which are obviously involved in its own transfer, so it was proposed that one of the *C. difficile* ICE mediated its transfer via a mechanism like Hfr in *E. coli* (Brouwer et al., 2013).

Two studies have deleted a prophage from *C. difficile* 630, lysogenic for two inducible prophages, ϕCD630-1 and ϕCD630-2 (Sebaihia et al., 2006; Goh et al., 2007). Hong *et al* deleted ϕCD630-2 using CRISPR-Cpf1 (Hong et al., 2018), and Peltier *et al* deleted ϕCD630-2 using a toxin-antitoxin system to select for double crossover mutants (Peltier et al., 2020). R20291 is a hypervirulent epidemic *C. difficile* strain that is well-characterized, and its genome was predicted to contain a complete prophage genome (Stabler et al., 2009), ϕ027, shown to spontaneously excise from the bacterial chromosome and circularize to exist extrachromosomally (Sekulovic and Fortier, 2015). ϕ027 has not been characterized as a functional phage, perhaps due to the lack of a suitable indicator strain for phage infection.

*C. difficile* possesses the type I-B CRISPR-Cas system utilizing several Cas proteins (Boudry et al., 2015; Andersen et al., 2016), which is different to the commonly used type II system utilizing a Cas9 or Cas12a protein. The use of heterologously expressed type II systems in *C. difficile* could avoid native type I-B CRISPR-Cas systems interference, which was successfully re-programmed for gene deletion in both *C. difficile* 630Δ*erm* and R20291 (Maikova et al., 2019). Indeed, native CRISPR-Cas systems of *C. difficile* 630 and R20291 (Boudry et al., 2015) did not interfere with several reports of successful gene deletants selected using Cas9 or Cas12a (McAllister et al., 2017; Hong et al., 2018; Wang et al., 2018; Ingle et al., 2019) expressed from pMTL8000 plasmids (Heap et al., 2009). In this study, we used a similar strategy of CRISPR-Cas9 to select deletants of Tn*5397* from *C. difficile* 630 and ϕ027 prophage from R20291(Stabler et al., 2009), allowing their contribution to *C. difficile* biology to be determined.

## 2 Materials and methods

### 2.1 Bacterial strains and growth conditions

The *C. difficile* and *Escherichia coli* strains used in this study are listed in Table 1. All bacterial strains were stored at −80°C in their respective medium [brain heart infusion broth (BHIB, Neogen, UK) for *C. difficile* and Luria-Bertani (LB, Neogen, UK) for *E. coli*] with 20% (v/v) glycerol. *C. difficile* agar cultures were freshly prepared weekly from −80°C stocks on Brazier’s agar (Neogen, UK) supplemented with 1% defibrinated horse blood (Thermo Scientific, UK), 250 μg/mL cycloserine and 8 μg /mL cefoxitin (Merck, UK) incubated anaerobically (Don Whitley DG250: 10% H_2_, 5% CO_2_, 85% N_2_) at 37°C for 2–3 days. *C. difficile* broth cultures were prepared from agar cultures either in BHI, BHI supplemented with 5 g/L yeast extract (Oxoid, UK) and 0.1% L-cysteine (Merck, UK) (BHIS), or BHIS supplemented with the following antibiotics/inducer when appropriate: thiamphenicol (Tm, 15 μg/mL, Merck, UK), D-cycloserine (250 μg/mL) and kanamycin (50 μg/mL, Merck UK), and incubated 16–18 h or BHIS agar supplemented with the following antibiotics/inducer when appropriate: 7% defibrinated horse blood, Tm (15 μg/mL), D-cycloserine (250 μg/mL) and kanamycin (50 μg/mL), and incubated 2–3 days. Log phase cultures were prepared from 1 mL of 16–18 h cultures in 10 mL pre-reduce BHIB incubated anaerobically for 4 h at 37°C. *E. coli* NEB® 5 -alpha or NEB® 10-beta (New England Biolabs or NEB, UK) was used as the general host for plasmid construction and gene cloning. *E. coli* CA434 was used as the donor for conjugation with *C. difficile*. Transformation of *E. coli* was carried out by heat-shock at 42°C for either 45 s (*E. coli* CA434) or 30 s (*E. coli* NEB® 5 -alpha or NEB® 10-beta), and transformants were selected on LB agar plates (Difco, UK) supplemented with 25 μg/mL chloramphenicol (Biological Life Science USA), and grown in LB broth (Neogen, UK) with 12.5 μg/mL chloramphenicol.

**Table 1:** Bacterial strains and plasmids used in this study.

**Organism/plasmid**	**Relevant features** ^**1**^	**Source and reference**
*C. difficile* R20291	Ribotype (RT) 027, MLST sequence type (ST) 1 (76) (Bletz et al., 2018), toxinotype III, lysogen of ϕ027 prophage (Stabler et al., 2009)	Brendan Wren, LSHTM CRG2021 lineage (Monteford et al., 2021)
*C. difficile* NCTC11207	RT 001, ST3, toxinotype 0, susceptible to ϕ027 infection	Melinda Mayer, Quadram Institute. Genome sequence determined in this study. BioProject PRJNA993731, Accession number CP129979.
*C. difficile* CD37	RT 009, ST03, non-toxigenic, Tet^S^ Erm^S^ Rif^R^	Smith et al. (1981) and Gawlik et al. (2015)
*C. difficile* 630	RT 012, ST54, toxinotype 0, Tet^R^ Erm^R^ Rif^S^	Sebaihia et al. (2006), Monot et al. (2011), and Riedel et al. (2015)
630Δ*erm tcdB*::*erm*(B)	*C. difficile* 630Δ*erm*::Δ*tcdB,* contains wild type Tn*5397*, Tet^R^ Erm^R^ Rif^S^	Kuehne et al. (2010)
*630*Δ*erm*::Δ*Conj*	630Δ*erm*::Δ*tcdB* containing a 5 kb deletion of the Tn*5397* conjugation region, Tet^R^ Erm^R^ Rif^S^	This study
*630*Δ*erm*::ΔTn*5397*	630Δ*erm*::Δ*Conj* that had lost Tn*5397*, Tet^S^ Erm^R^ Rif^S^	This study
*630*Δ*erm*::*tcdB^−^* Δ*Conj*	Transconjugant from the mating of *630*Δ*erm*::ΔTn*5397* and CD37. Tet^S^ Erm^R^ Rif^R^	This study
68P10-23/68P10-30	*C. difficile* R20291Δϕ027, Tm^S^ prophage deletants	This study
NEB® 10-beta	High efficiency competent *E. coli* DH10β from NEB. Δ(*ara-leu*) 7697 *ara*D139 *fhuA* Δl*acX74 gal*K16 *gal*E15 *e14*- ϕ80*dlacZ* M15 *rec*A1 *rel*A1 *end*A1 *nup*G *rps*L (StrR) rph spoT1 Δ(*mrr-hsd*RMS-*mcr*BC)	New England Biolabs, UK
NEB® 5-alpha	Competent *E. coli* DH5α derivative from NEB. *fhuA2*Δ(*argF-lacZ*)U169 *phoA glnV44* Φ80Δ(l*acZ*)M15 *gyrA96 rec*A1 *rel*A1	New England Biolabs, UK
*E. coli* CA434	HB101 carrying the IncP conjugative plasmid R702	Williams et al. (1990) and Purdy et al. (2002)
pMTL83151	replicon of pCB102, *catP*, *colE1*, *traJ*	Heap et al. (2009)
pPM100	pMTL83151 modified to include P_xyl_/tetO, Cas9, P_tetM_, gRNA scaffold, *lacZ*. This is the basic modular vector that can be used to manipulate *C. difficile* (Figure 1).	This study
pPM101	pPM100 containing sequences encoding gRNA targeting region B (Figures 1, 2).	This study
pPM102	pPM100 containing sequences encoding gRNA targeting region C (Figures 1, 2).	This study
pPM103	Contains LHA and RHA1 (Figure 2) cloned into pPM101	This study
pPM104	Contains RHA1 and RHA2 (Figure 2) cloned into pPM102	This study
pAN721	gRNA_1040 targeting the coding strand nt 76..95 of ϕ027 integrase gene (CDR20291_1415) with a PAM of tgg, cloned into pPM100	This study
pAN821	1 kb (HA1) cassette of homology arms consisting of 500 bp of sense sequences flanking the ϕ027 integrase gene (CDR20291_1415) of the circularized phage genome, cloned into pAN721	This study

^1^Antibiotics are abbreviated as follows: erythromycin (Erm), thiamphenicol (Tm), tetracycline (Tet).

**Figure 1:**
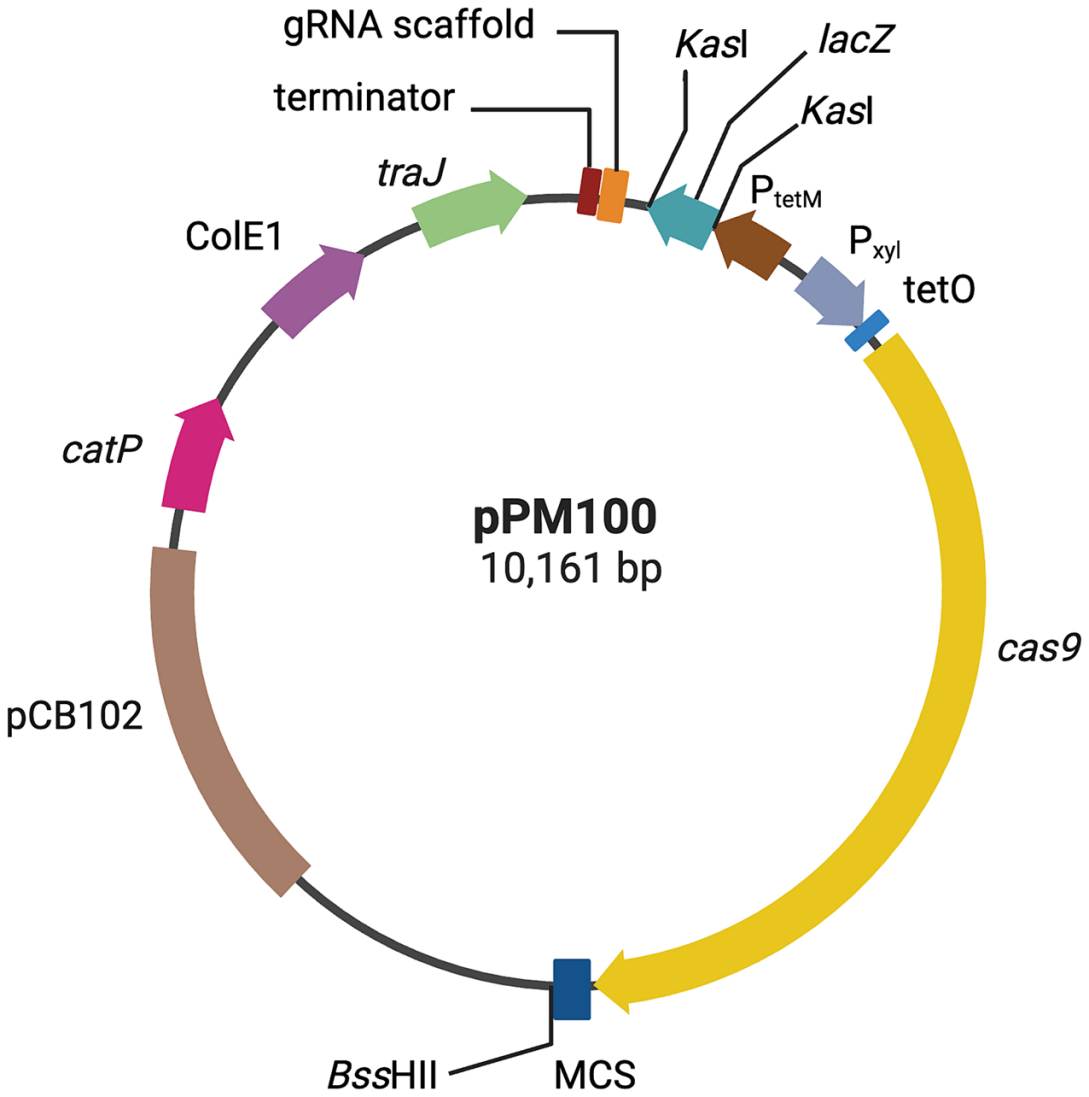
CRISPR-Cas9 genome editing vector. Plasmid map representation of the CRISPR-Cas9 vector developed in this study, pPM100. The *S. pyogenes cas9*, is under the control of the Pxyl-tetO promoter; a gRNA scaffold, consisting of a gRNA handle under the control of *C. difficile* P_tetM_ promoter, and a multiple cloning site (MCS) for cloning of an editing template, containing upstream and downstream chromosomal regions flanking a deletion target site. The editing regions are cloned into the MCS (see text for more details). Created with biorender.com.

**Figure 2:**
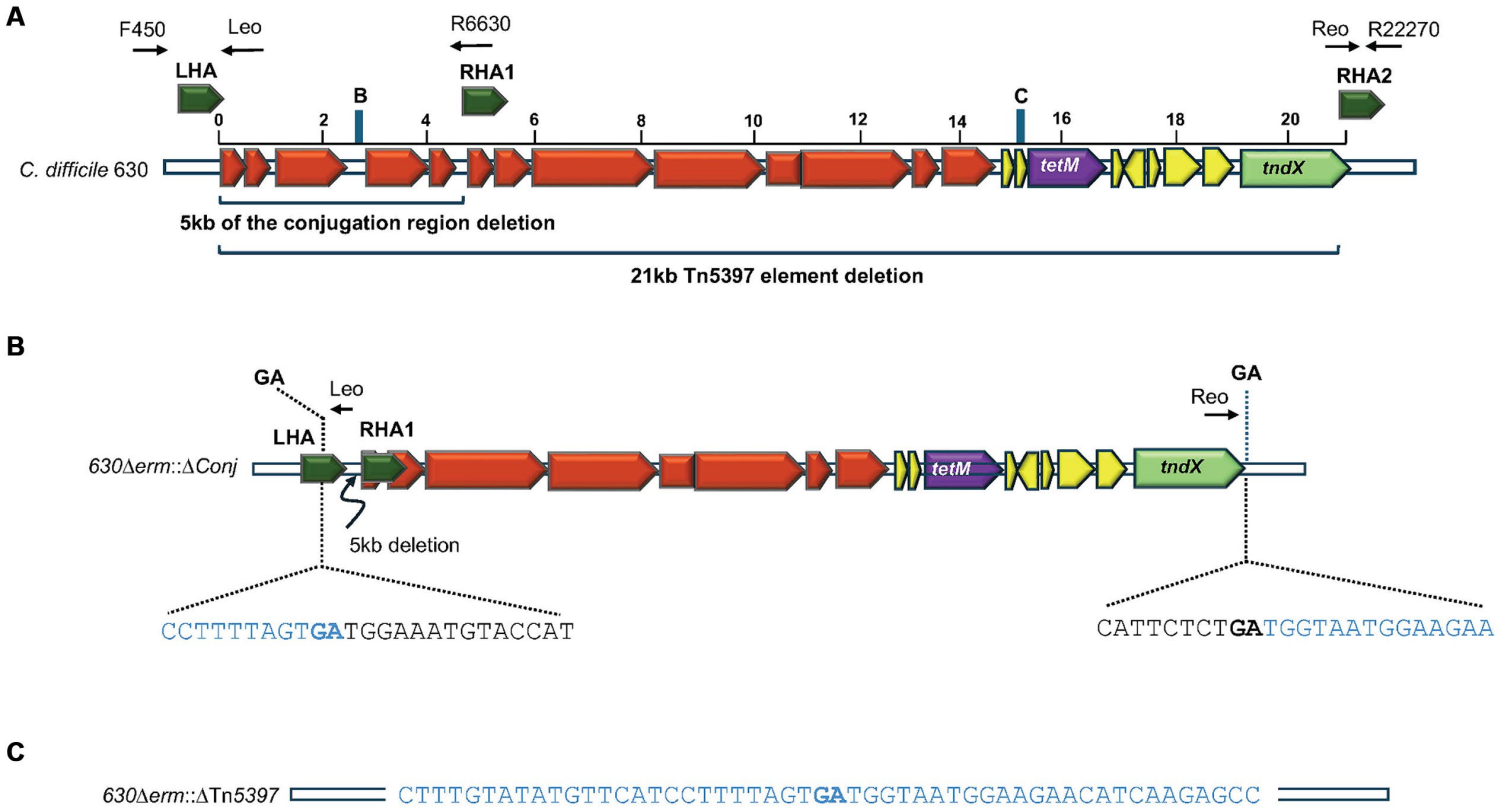
Deletion of Tn*5397* from *C. difficile* 630. **(A)** Schematic representation of *C. difficile* Tn*5397* conjugative transposon sequence. Black arrows above the figure represent binding sites of primers used in PCR analysis, the sequences of these are shown in Supplementary Table S1. The conjugation region genes are shown in red, the *tetM* gene is shown in purple, regulatory genes are shown in yellow, excision/insertion (*tndX*) shown in green. Brackets indicate the 5 kb conjugation region deleted from Tn*5397,* and the whole 21 kb Tn*5397.* Homology arms flanking regions for deletion are in deep green. Blue bars represent the positions of 20-nt sgRNA retargeting sequences within the region targeted for deletion from Tn*5397* labelled B and C, corresponding to the sequences in Supplementary Table S2. **(B)** *630*Δ*erm*::Δ*Conj* carrying the 5 kb deletion and sequence at the ends of the remaining element. Bacterial sequences are in blue, Tn*5397* sequences are in black, GA in bold is the junction between the element. **(C)** Regenerated DNA sequences of *630*Δ*erm::*ΔTn*5397* after deletion of whole element.

Growth curves of *C. difficile* R20291 and R20291Δϕ027 were generated from OD_600 nm_ readings over 18 h in 96-well plates. Microtiter plates, sealing films, BHIB and *C. difficile* minimal medium (CDMM) (Cartman and Minton, 2010) were pre-reduced before use. Bacterial cultures in BHIB of 18 h were anaerobically diluted in growth media to OD_600 nm_ of 0.1, and 200 μL volumes distributed into triplicate wells (3 technical repeats). Growth media alone were similarly distributed, serving as blanks and negative controls. The 96-well microtiter plate was sealed anaerobically, then transferred to a microtiter plate reader set at 37°C and kinetic measurements taken for 18 h every 15 min after 5 s of agitation. The experiment was repeated four times from which average OD_600 nm_ values and standard deviation were calculated in Microsoft Excel and plotted in Prism 10 (GraphPad).

### 2.2 Phage induction, propagation, and purification

To induce ϕ027, known to exist within *C. difficile* R20291 (Stabler et al., 2009), a 16–18 h culture of *C. difficile* R20291 in 10 mL BHIB was treated with 3 μg/mL of mitomycin C (Merck, UK) for 6 h at 37°C. The induced culture was centrifuged at 4500 x g for 15 min and the supernatant was filtered through a sterile 0.45 μm membrane filter (Fisher Scientific, UK). Plaque assays were carried out in anaerobe basal agar (Oxoid, UK) with 600 μL of 4 h log phase cultures of NCTC11207 in BHIB and 100 μL of R20291 filtrate as previously described (Goh et al., 2005). A no-phage control was included with every plaque assay to ensure no spontaneously induced prophages from NCTC11207 were co-cultivated with ϕ027. Two rounds of single plaque propagation were carried out on NCTC11207, followed by whole plate assays for phage propagation to obtain crude phage suspensions as previously described (Goh et al., 2005). Crude phage suspensions were treated with DNase I (2 U/μL, Merck, UK) and RNase A (10 μg/mL, Merck, UK), precipitated by 1 M NaCl and 10% w/v PEG 8000, and recovered with chloroform to yield semi-pure suspensions. These were then either purified through a pre-formed CsCl density gradient of 1.3 g/mL, 1.5 g/mL, and 1.7 g/mL at 60,000 x *g* for 2 h at 4°C (Sorvall WX 80+ Ultracentrifuge, AH 650 swing out rotor) and dialyzed as previously described (Sambrook and Russell, 2001a) to yield purified suspensions, or concentrated by ultrafiltration through Amicon Ultra-15 3 kDa MWCO Centrifugal Filter Devices (Merck, Germany) spun at 4,000 × *g* for 60 min at room temperature.

### 2.3 Bacterial, phage, and plasmid DNA extraction

Five milliliters of a 16–18 h *C. difficile* broth culture was pelleted and frozen at −20°C before genomic DNA was extracted using the GenElute Bacterial Genomic DNA Kit (Sigma-Aldrich, UK). Phage DNA was extracted from semi-purified or purified dialyzed phage suspensions using either phenol:chloroform:isoamyl alcohol (Invitrogen, UK) (Sambrook and Russell, 2001b) or Phage DNA Isolation kit (Norgen Biotek, Canada). Plasmid DNA was extracted from 1 mL of 16–18 h *E. coli* broth cultures grown aerobically with agitation at 200 rpm using either the plasmid miniprep kit (Qiagen, UK) or Monarch Plasmid Miniprep kit (NEB UK).

### 2.4 PCR of phage, and phage and bacterial attachment sites in *Clostridioides difficile* R20291

Primer sets LCF889/LCF 890 and LCF 887/LCF 888 from Sekulovic and Fortier (2015) were used to confirm phage attachment site for ϕ027 in R20291 (Supplementary Table S1). Six primer sets specific for ϕ027 *orf 1415, 1416, 1417, 1418, 1419,* and *1464a* were used to confirm the presence of the prophage in R20291 (Supplementary Table S1). OneTaq DNA polymerase and reaction buffer (NEB UK) were used for PCR reactions according to cycling conditions recommended by the manufacturer.

### 2.5 Construction of plasmids for ICE and prophage deletion

A modular vector pPM100, i.e., where desired DNA modules can be inserted in a single step, was constructed (Table 1 and Figure 1). The starting point was the *E. coli–C. difficile* shuttle vector pMTL83151, which contains the origin of replication from plasmid pCB102 (this replicon is unstable in *C. difficile* allowing it to be used as conditional lethal vector), along with the *catP* selective marker, the ColEI replicon and the *mob* region from RK2 (Heap et al., 2009). The Cas9 gene cassette of *Streptococcus pyogenes* from pCas9 (Addgene, UK) and the inducible promotor P*_xyl/tetO_* from pRPF185 (Fagan and Fairweather, 2011) were used as templates for PCR utilizing Pxyl/tetO-F with Pxyl/tetO-R and Cas9-F with Cas9-R primer pair, respectively. The two fragments were fused using splicing by overlap extension (SOE) PCR utilizing Pxyl/tetO-F and Cas9-R primer pair. The Pxyl/tetO-Cas9 fragment was cloned into pMTL83151 upstream of the Fdx terminator between *Xma*I and *Sal*I restriction enzyme (Thermo Scientific, UK) sites resulting in plasmid pPM100. For deleting Tn*5397* from *C. difficile* 630, the sgRNA encoding fragment was synthesized by Thermo Fisher Scientific and consisted of: the strong Tn*916* derived promoter (P*_tetM_*) (Su et al., 1992) and a 20 bp gRNA targeting sequence that was selected using an algorithm for scoring and ranking potential target sites with the Benchling CRISPR design tool^1^ (Supplementary Table S2). The sgRNA fragment was annealed by heating for 5 min then cloned into pMTL83151-CRISPR-Cas9 (pPM100) upstream of CD0164 terminator between *Xma*I and *Not*I sites. The editing regions were amplified by PCR using two pairs of primers (Supplementary Table S1) to produce fragments homologous to sequences targeted for recombination, and they were cloned next to the multiple cloning site (Figures 1, 2). Individual editing fragments were then fused together by SOE PCR at the *Bss*HII site resulting in plasmid (pPM103 and pPM104) respectively (Table 1 and Figure 2).

For deleting ϕ027 from *C. difficile* R20291, a gRNA sequence targeting the region of interest identified by Benchling was chosen to which pairs of self-complementary oligos (Supplementary Tables S1, S2) were annealed at 50 pmol/μL in annealing buffer (10 mM Tris pH 8, 50 mM NaCl, 1 mM EDTA pH 8), phosphorylated with 15 U T4 polynucleotide kinase (NEB, UK), ligated to *Kas*I-linearized (10 U of SspD1, Thermo Scientific, UK) and gel-extracted plasmid (Monarch kit, NEB, UK) with 5 U of T4 DNA ligase (Thermo Scientific, UK), then transformed by chemically competent NEB® 5-alpha cells. Cloned gRNA was confirmed by PCR using OneTaq DNA polymerase (NEB, UK) and Sanger sequencing (Source Biosciences Ltd., UK) with primers pMTL83151bb_99 and PtetM_191 (Supplementary Table S1). NEBuilder® HiFi DNA Assembly Tool designed primers for amplification and assembly of homology arms (Supplementary Table S1). Phusion High-Fidelity DNA Polymerase (Thermo Scientific, UK) amplified 0.5 kb sequences flanking the ϕ027 integrase gene with primer pairs int_RLA_rev/phi027_1415_LHA_R and int_RLA_fwd/ phi027_1415_RHA_F. Amplicons were cloned with NEBuilder® HiFi DNA Assembly Master Mix (NEB) into *Bss*HII linearized and gel-extracted pAN721. Assembled plasmids were transformed by heat-shock to NEB® 10-beta to generate pAN821. Cloned inserts were Sanger sequenced (Source Biosciences Ltd., UK) with primer pairs pHHCas9_HACS_F/R, and 14152HA_pwalk1/2 (Supplementary Table S1).

### 2.6 Mating experiments

#### 2.6.1 Filter-mating experiments between *Clostridium difficile* 630 and CD37

To test for PaLoc and Tn*5397* transfer, methods described in Brouwer et al. (2013) were used with the following modifications. *C. difficile* was grown in BHIB for 18 h anaerobically, then subcultured to fresh broth at 37°C until mid-exponential phase (OD_600 nm_ of 0.45). Cultures of *C. difficile* 630 (Tet^R^ Erm^R^ Rif^S^) or 630Δ*erm tcdB*::*erm*(B) (Tet^R^ Erm^R^ Rif^S^) donors and CD37 (Tet^S^ Erm^S^ Rif^R^) recipient were mixed, and 200 μL was spread on nitrocellulose 0.45 μm pore-size filters on BHI agar and incubated for 18 h at 37°C in an anaerobic environment. The filters were removed from the agar plates and placed in 20 mL bottles and vigorously washed with 1 mL BHIB. Aliquots (100 μL) were spread on BHI agar supplemented with either 10 μg/mL tetracycline, 100 μg/mL erythromycin, 15 μg/mL thiamphenicol, or (25 μg/mL) rifampicin and incubated anaerobically for 48 h. Putative transconjugants were subcultured on fresh selective plates and incubated for a further 48 h. Selection of transfer of Tn*5397* from 630Δ*erm tcdB*::*erm*(B) to CD37 was made on plates containing rifampicin and tetracycline. Transfer of the PaLoc from 630Δ*erm tcdB*::*erm*(B) and strains that had lost Tn*5397,* or contained a deletion of part of the conjugation region, was made on plates containing erythromycin and rifampicin.

#### 2.6.2 Transfer of plasmids from *Escherichia coli* to *Clostridium difficile*

Single colonies of *E. coli* CA434 containing mutagenic plasmids were grown anaerobically overnight at 37°C in pre-reduced BHIS with 12.5 μg/mL chloramphenicol, 1 mL was pelleted and washed in pre-reduced BHIS. Two hundred microliters of overnight *C. difficile* culture was heated to 52°C for 5 min, cooled for 2 min, (the heating step was only required when R20291 was the recipient) then mixed with *E. coli* donor cell pellets and incubated for 8 h as described previously (Kirk and Fagan, 2016). The mating mixture was spotted onto BHIS agar, grown anaerobically for 8 h, harvested in 1 mL pre-reduced phosphate-buffered saline (PBS) and plated onto BHIS with 250 μg/mL cycloserine, 50 μg/mL kanamycin, and 15 μg/mL Tm (CKTm). After 24–48 h of growth, colonies were picked and transfer of mutagenic plasmid pPM103, pPM104 or pAN821 was confirmed by PCR (see Supplementary Table S1 and the results section for more details). *C. difficile* transconjugants were re-streaked onto BHIS agar with appropriate antibiotics. After 2 days, a single colony was inoculated into pre-reduced 10 mL BHIS supplemented with appropriate antibiotics and grown overnight for gDNA extraction for further PCR confirmation. Conjugation frequency was calculated against either donor or recipient in mating mixtures. Donor and recipient cultures were serially diluted 10-fold in pre-reduced PBS and plated onto LB and BHI plates, respectively. Colonies were counted after 24 h anaerobic incubation for *E. coli* and 48 h incubation for *C. difficile*. The conjugation frequency was calculated as colony forming units (CFU) of transconjugants/CFU of donor or recipients.

### 2.7 Induction of CRISPR-Cas9 system and MGE deletion

*Clostridioides difficile* containing mutagenic plasmid on BHIS CKTm and 7% defibrinated horse blood plates were grown in 10 mL BHIB with Tm (15 μg/mL) for 16–18 h, serially diluted 10-fold in pre-reduced sterile 1 x PBS, spread-plated onto dried and pre-reduced BHIS + anhydrotetracycline (aTC, Merck, UK, 30 ng/mL) + Tm (15 μg/mL) plates and grown for 2 days. Five to 10 colonies were screened by colony PCR for ϕ027 prophage or Tn*5397* deletion. For prophage deletion primer pairs phiR2_1415_F/phiR2_1,415_R, phi027_1464a_F/phi027_1464a_R, LCF887/LCF889, LCF888/LCF890 and catP_2/3 were used (Supplementary Table S1). To confirm Tn*5397* conjugation region deletion, primers Tn*5397*(F450) and Tn*5397*(R6630), and Tn*5397*(R450) and Tn*5397*(R22270) were used to determine if the whole element was absent (Supplementary Table S1).

### 2.8 Plasmid curing

To cure the plasmid from strains with mutant or deleted target MGE (ϕ027 or Tn*5397*), a single colony of the mutant with the desired deletion (i.e., deletant) was subcultured in BHIB with no antibiotics. After 18 h, 100 μL of the culture was used to inoculate 10 mL of BHIB. This subculture was repeated daily for up to 10 days. Ten-fold serial dilutions of deletant culture in pre-reduced 1 x PBS were made after each subculture for Tn*5397* deletants, but only at the end of 10 days for ϕ027 deletants, and 100 μL of the 10^−5^ dilution was spread onto BHI agar plates without antibiotic. Replica plating was performed on agar supplemented with 15 μg/mL Tm, tetracycline (Tet) or erythromycin (Erm) to identify colonies that have lost the mutagenesis plasmid (i.e., either pPM103, pPM104 or pAN821). Colonies that had lost antibiotic resistance at the least number of subcultures were isolated for further study to avoid excessive subculture. Loss of the plasmid was confirmed by PCR using plasmid-specific primers (Cas9-F and Cas9-R, or catP_2/3) (Supplementary Table S1). For Tm sensitive (Tm^S^) prophage deletants putatively cured of plasmid, gDNA was extracted and checked with 1464a_F/R, NF1643/44, and catP_2/3 primers, then sequenced by Illumina sequencing. To screen for further loss of a truncated Cas9 plasmid remnant in sequenced Tm^S^ prophage deletant strains, single colonies were picked for colony PCR using primers 68P1023_LF/LR, specific for the left junction of the integrated plasmid remnant (Supplementary Table S1). Putatively negative colonies were grown in BHIB for gDNA extraction and confirmation by PCR with the same primers in addition to 68P1023_RF/RR, and pHHCas9_3F/end.

### 2.9 Genomic DNA library preparation for Nanopore sequencing

For long-read Oxford Nanopore Technology (ONT, UK) sequencing of NCTC11207, 5 mL of 18 h culture was pelleted and frozen at −20°C before DNA extraction using the Qiagen MagAttract High Molecular Weight DNA Kit (Qiagen, UK). DNA quality and quantity were assessed using NanoDrop, Qubit (Thermo Fisher Scientific, UK), and Agilent TapeStation instruments (Agilent, UK). ONT sequencing libraries were prepared by multiplexing DNA from *C. difficile* isolates per flow cell using a Nanopore protocol for native barcoding of genomic DNA (version NBE_9065_v109_revAC_14Aug2019). This firstly involved DNA repair and end-prep carried out with NEBNext FFPE DNA Repair Mix (M6630, NEB, UK), NEBNext Ultra II End repair/dA-tailing module (E7546, NEB, UK), and AMPure XP beads (Beckman Coulter, UK). Secondly, native barcode ligation was carried out using the Native Barcoding Expansion kit (EXP-NBD104; ONT, UK) and NEB Blunt/TA Ligase Master mix (M0367, NEB, UK). Thirdly, adapter ligation using Ligation Sequencing Kit (SQK-LSK109, ONT, UK), NEBNext Quick T4 DNA Ligase (E6057, NEB UK), NEBNext Quick Ligation Reaction Buffer (B6058, NEB, UK). Sequencing libraries were loaded onto a R9 generation flow cell (FLO-MIN106) and sequenced in MinION Mk1C (ONT, UK), stopping after 25 h.

### 2.10 Nanopore sequence analysis of NCTC11207

Before assembly, long read sequences were filtered using Filtlong v0.2.0, keeping the minimum length of 1,000 bp and 90% of best quality sequences^2^. Genome assembly was performed using Flye assembler v2.9 (Kolmogorov et al., 2019) and Trycycler v0.5.0 (Wick et al., 2021), with a standard protocol recommended by the developers (Wick et al., 2021). The final assembly graph was visualized and polished with Bandage v0.8.1 (Wick et al., 2015). Multi-Locus Sequence Typing was performed *in silico* using BIGSdb v1.32.0 hosted at PubMLST^3^ and the scheme of Griffiths et al. (2010). Prophage screening of the NCTC11207 chromosome was performed using PHASTER (Arndt et al., 2016). The final circular genome was annotated using the NCBI Prokaryotic Genomes Annotation Pipeline (PGAP) (Tatusova et al., 2016) and is now available in GenBank under BioProject PRJNA993731 (accession number CP129979). Similarity of the ϕ027 genome to genomes of NCTC11207 prophages 1 and 2 was determined using VIRIDIC (Moraru et al., 2020).

### 2.11 Illumina sequencing of ϕ027 deletants and deletion/insertion confirmations

*Clostridioides difficile* gDNA was extracted using either GenElute Bacterial kit (Merck, UK) or Qiagen MagAttract High Molecular Weight DNA Kit (Qiagen, UK), checked for quality, and paired-end sequenced at Microbes NG (UK, 2 × 250 bp, 30 x coverage) or SeqCenter (USA, 2 × 151 bp, 30 x coverage). Trimmed Illumina reads facilitated by these vendors were mapped against the genome of *C. difficile* R20291 (accession number NZ_CP029423.1). Unmapped reads were afterwards *de novo* assembled using Unicycler v0.4.8 and mapped against pAN821 using the BWA-MEM algorithm (arXiv:1303.3997v2). To detect ORF 1465 in deletant and WT by PCR, primers LF1 and RR1 (Supplementary Table S1) were used on two batches of genomic DNA prepared from WT and deletant cultures as described in 2.3. OneTaq DNA polymerase and reaction buffer (NEB UK) were used for PCR reactions according to cycling conditions recommended by the manufacturer.

## 3 Results

### 3.1 Construction of a modular vector for gene knock out in *C. difficile*

A simple modular vector, pPM100, was constructed for generating CRISPR-directed mutations in *C. difficile* (see Materials and Methods and Figure 1). This plasmid is unstable in *C. difficile* and therefore an ideal delivery vector. Furthermore, all the modules on this vector can be easily replaced or modified making it a useful and efficient tool for relatively easy manipulation of *C. difficile.*

### 3.2 Editing regions designed to delete the conjugation region of Tn*5397* resulted in a mixture of clones some of which had lost just the conjugation region and some the whole of Tn*5397*

To investigate the role of Tn*5397* in genome mobility and to generate a Tet sensitive (Tet^S^) derivative of 630Δ*erm tcdB*::*erm*(B) [this has a Clostron insertion conferring Erm resistance (Erm^R^) in the *tcdB* gene to allow for the selection of PaLoc transfer (Kuehne et al., 2011; Brouwer et al., 2013)], we initially wanted to precisely delete the whole of Tn*5397.* This was attempted by generating a CRISPR-Cas9 vector (pPM103) with gRNA encoding sequences targeting region C of Tn*5397* (Figure 2A and Supplementary Table S2) and editing regions flanking the insertion site of Tn*5397* in 630Δ*erm tcdB*::*erm*(B) (LHA and RHA2, Figures 1, 2A). This plasmid was conjugated into 630Δ*erm tcdB*::*erm*(B) and the resulting four Tm-resistant (Tm^R^) transconjugants were subject to Cas9 induction and then screened by PCR for loss of Tn*5397* using primers flanking Tn*5397*. All four transconjugants still had Tn*5397*. It was assumed that the reason for this failure was that the region we were trying to delete is too large. Therefore, it was decided to delete part of the conjugation region. To do this, the CRISPR-Cas9 vector (pPM104) containing a gRNA encoding region targeting region B at 2500 bp on Tn*5397* and an editing region consisting of LHA and RHA1 was used (Figure 2A and Supplementary Table S2). Plasmids were transferred by conjugation to 630Δ*erm tcdB*::*erm*(B), and five Tm^R^ colonies arose. These were subject to PCR with primers F450 and R6630 and these yielded a product of 1.5 kb (no product was obtained with strains carrying wild-type Tn*5397* presumably because the product was too large) (Figure 2A). DNA sequence analysis of this product confirmed that a precise 5 kb deletion of part of the conjugation region had occurred (Figure 2B and Supplementary Figure S2).

One of the strains, *630*Δ*erm::*Δ*Conj,* carrying the 5 kb deletion was selected for further study. It was grown for 18 h in drug free broth then plated onto drug free media, 600 colonies were screened for loss of resistance to Tet, and 2 of 600 were sensitive, hence the mutation efficiency was 0.3%.

PCR analysis of the two tetracycline-sensitive mutants using primers flanking Tn*5397* (F450 and R22270 in Figure 2A) gave a product of 1.2 kb (no product was obtained in strains carrying wild-type Tn*5397* or those carrying the 5 kb deletion). One of these strains was selected for further study and designated *630*Δ*erm*::ΔTn*5397.* DNA sequencing of the PCR product showed that the target site of Tn*5397* had been regenerated (Figure 2C) and that the whole of the transposon had been lost. This implies that deletion of part of the conjugation region destabilizes Tn*5397* so that it can still excise from the host chromosome and circularize but presumably due to the large deletion some circular molecules are lost. This idea was supported by the fact that we could detect the presence of a circular form of the element using primers Tn*5397* (Leo) and Tn*5397* (Reo) in the *630*Δ*erm*::Δ*Conj* mutants that contained the 5 kb deletion. These primers read out from the ends of Tn*5397* and will only form a product when the ends are ligated together in a circular form of the element (Supplementary Figure S1 and Figure 2A). Diagrams showing these events in wild-type Tn*5397* have been previously published and are summarized in Supplementary Figure S1 (Wang et al., 2000; Wang and Mullany, 2000; Brouwer et al., 2011). No product was obtained from the tetracycline sensitive strains.

### 3.3 Strains that have lost Tn*5397* can still transfer the PaLoc at the same frequency as WT

Tn*5397* is the nearest MGE to the PaLoc in the 630Δ*erm tcdB*::*erm*(B) chromosome (Brouwer et al., 2013) and it was proposed that this element might be responsible for its mobilization. However, mutants that contain a deletion of the conjugation region (*630*Δ*erm*, Δ*Conj*) and those that have lost Tn*5397* completely (*630*Δ*erm*, ΔTn*5397*) both transfer the PaLoc at the same frequency of around 1 × 10^−7^ of *erm^R^* transconjugants per donor (encoded by the Clostron inserted in the *tcdB* gene) as the WT contains an intact Tn*5397*. This shows that a genetic element other than Tn*5397* is responsible for PaLoc transfer, although we cannot completely rule out a role for this element.

### 3.4 Ф027 is a functional phage integrated in R20291

ϕ027 was first identified as a putative prophage integrated into the chromosome of *C. difficile* R20291 (GenBank accession numbers FN545816.1 and CP029423.1) (Stabler et al., 2009). This phage and its bacterial attachment sites, *attP* and *attB,* were later identified by PCR (Sekulovic and Fortier, 2015), as there was a population of spontaneously excised and re-circularized ϕ027 genomes in DNA preparations of the lysogen. In this study, we firstly confirmed R20291 was PCR positive for 6 predicted genes of ϕ027 (Supplementary Figure S3). Then we found that ϕ027 in *C. difficile* R20291 of CRG2021 lineage (i.e., closest to the original R20291 clinical isolate and less amenable to conjugation) (Monteford et al., 2021) is a functional and inducible phage that can be propagated on *C. difficile* NCTC11207 (GenBank accession CP129979), obtaining yields of 10^8^–10^9^ plaque forming units (pfu)/mL. NCTC11207 is a ribotype (RT) 001 strain (Table 1), which was sequenced here and predicted to contain two intact prophages whose features are summarized in Supplementary Table S3. To ensure that NCTC11207 prophages were not co-propagated with ϕ027, a phage buffer (i.e., no phage) control was included with every batch of propagated phage to ensure no plaques were derived from spontaneously induced NCTC11207 prophages. ϕ027 virion DNA was extracted and used for PCR to confirm the *attP* sequence 5′ tattacaacttaagtaaata 3′, is as previously found in circularized ϕ027 prophage DNA within R20291 (Sekulovic and Fortier, 2015). The linear phage DNA annotation is re-arranged to convention in Supplementary Figure S4 and Supplementary Table S4. We also obtained similar PCR results when using R20291 bacterial genomic DNA, confirming previous observations that ϕ027 spontaneously excises, and exists extra-chromosomally and as an integrated prophage located at nt. 1670843…1,726,837 encompassing CDS CDR20291_1415 to CDR20291_1464a (Genbank accession number FN545816.1 and Supplementary Figure S4; Sekulovic and Fortier, 2015).

**Figure 3:**
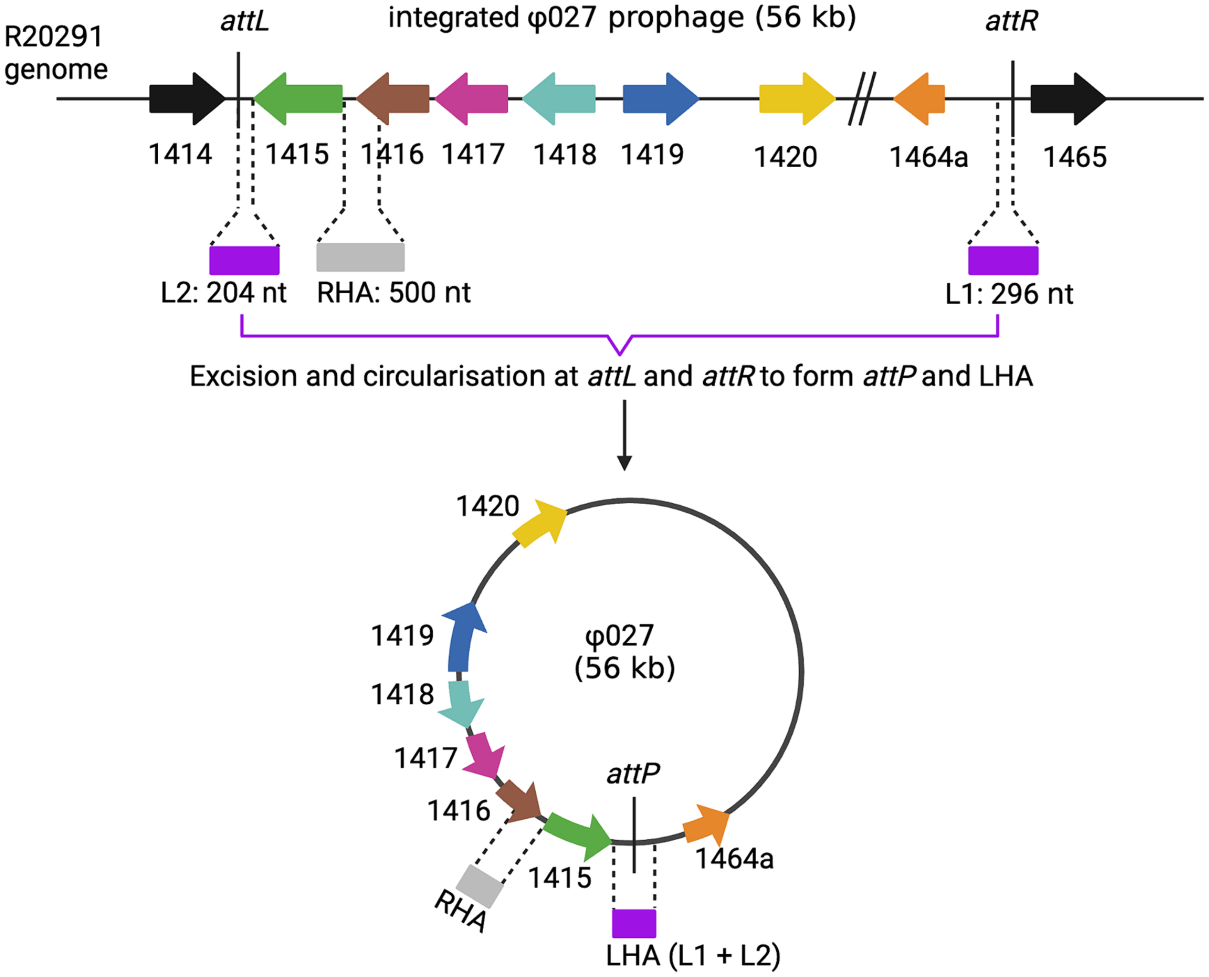
Homology arms for prophage deletion. The top line shows the integrated form of ϕ027, the junction between phage and bacterial DNA is shown at *attL* and *attR*. The phage can excise and circularize by site-specific recombination between *attL* and *attR* to generate a circular molecule with *attP* at the joint of the circular form. The location of homology arms that were used in mutagenesis experiments are shown. Note that LHA is only present at the joint of the circular form as it is composed of *attP*. Created with biorender.com.

As ϕ027 prophage spontaneously excises and circularizes within host cells, we hypothesized that removal of the integrase gene (CDR20291_1415) from the circular form would lead to loss of the phage as the circular form would not be able to reintegrate (Figure 3).

The conjugation frequencies of pPM100 (the plasmid backbone), pAN721 (targeting integrase and not containing homology arms), and pAN821 (targeting integrase and containing the 1 kb homology arms for integrase deletion) are shown in Supplementary Table S5. Conjugation frequencies of cells harboring pPM100 and pAN721 were comparable to those of cells harboring pAN821, indicating *cas9* expression is likely to be repressed in the absence of the inducer and did not affect cell viability.

### 3.5 Generation of *Clostridioides difficile* R20291 ΔФ027

**Figure 4:**
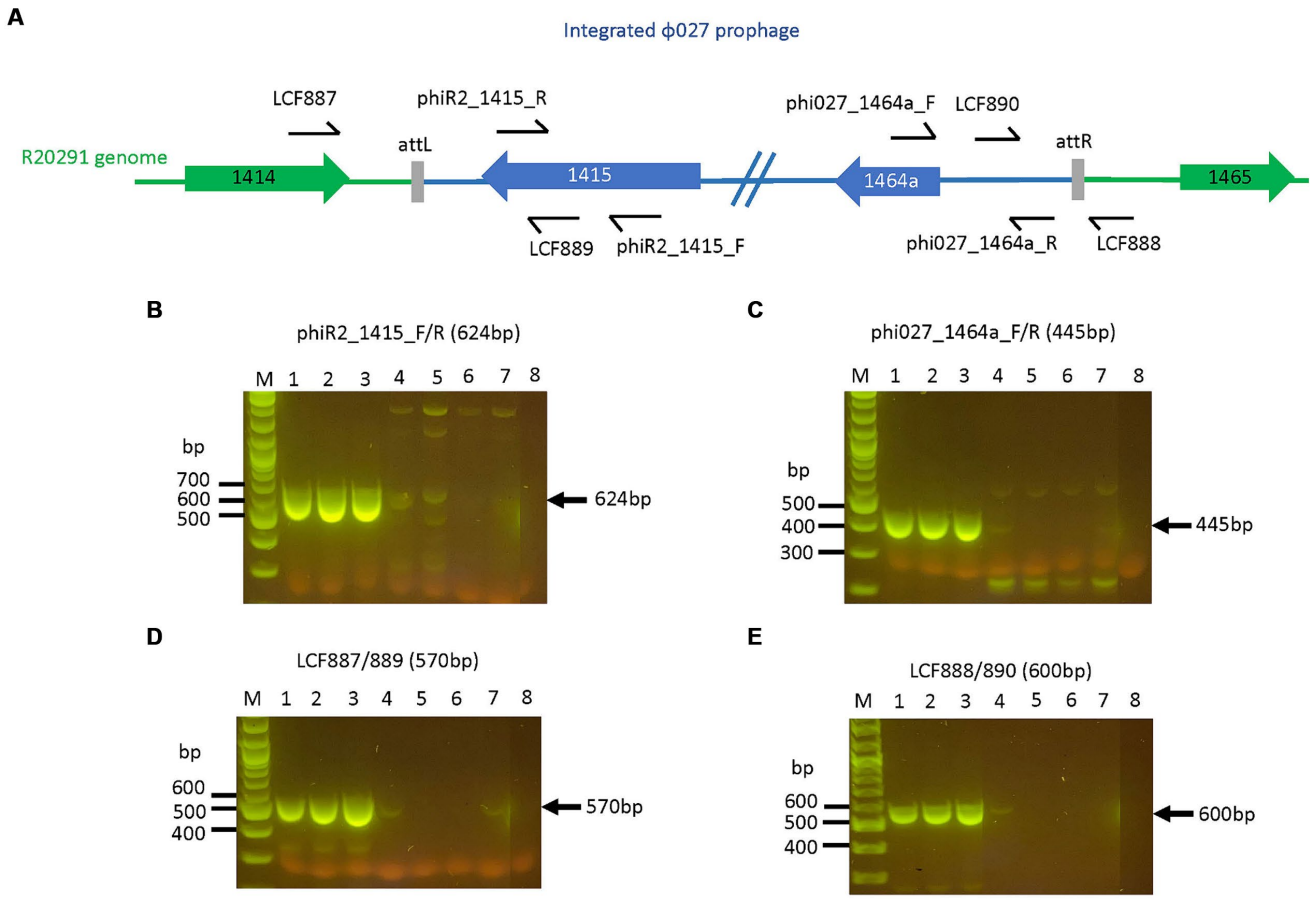
R20291 prophage deletants devoid of prophage features. Primers targeting selected features of the prophage are shown in **(A)**. The loss of **(B)** prophage *orf 1415* (integrase), **(C)** *orf 1464a*, **(D)** *attL*, and **(E)** *attR* were observed in R20291 deletants. Lane 1: WT R20291, 2: transconjugant of pPM100, 3: transconjugant of pAN721, 4–7: transconjugants of pAN821, 8: No template control. M: 1 kb Plus DNA ladder (NEB, UK).

**Figure 5:**
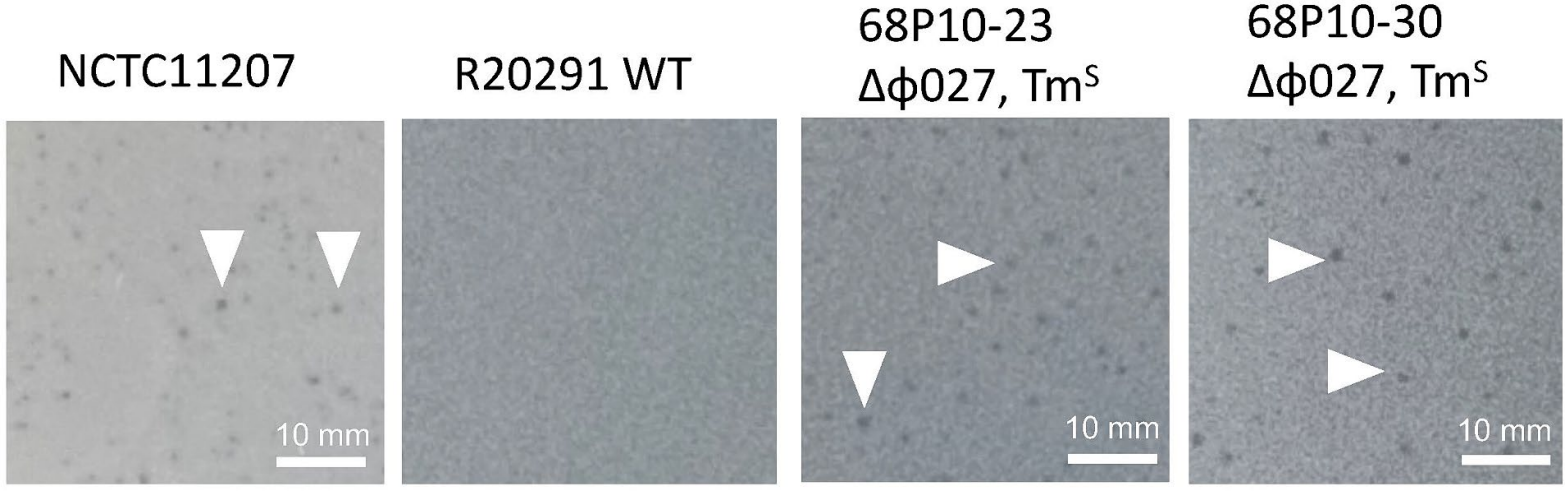
Phage susceptibility of R20291 prophage deletants. Susceptibility to ϕ027 infection was determined by plaque assays of Tm^S^ prophage deletants (68P10-23 and 68P10-30) that had lost plasmid-encoded resistance, the indicator strain NCTC11207 which is susceptible to ϕ027, and the lysogen WT R20291, which is resistant to ϕ027. White arrowheads indicate plaques, which vary in size and clarity.

Four of 8 transconjugants containing pAN821 with intact sgRNA, *cas9* and deletion cassette (Supplementary Figure S5) were devoid of prophage after aTC induction of Cas9 (Figure 4), could not be induced by mitomycin C to form plaques, and were susceptible to reinfection by ϕ027. The mutation efficiency was 50%. Curing of pAN821 from prophage deletants was attempted by passaging in non-selective BHIB for 10 days, then screening colonies for loss of susceptibility to Tm. Cultures containing control plasmids pPM100 (the plasmid backbone) and pAN721 (targeting integrase and not containing homology arms) were also screened for plasmid curing in the same way (Table 2). Just 1.5% (2/137) of colonies from the culture containing pAN821 had lost plasmid-encoded resistance after 10 passages. In contrast, plasmids lacking homology arms were all rapidly cured in this time from R20291 (Table 2). It is possible the homology arms allowed pAN821 to survive by recombining with the host genome. The actual mechanism for this requires further investigation. Susceptibility of two cured prophage deletants to ϕ027 infection was confirmed (Figure 5).

**Table 2:** Colonies screened for loss of mutagenesis plasmid.

Transconjugants	Growth on replica plates		Tm^S^	*catP* positive
	BHI	BHITm^1^		
pPM100	105	0	105	0
pAN721	130	0	130	0
pAN821	137	135	2	135

^1^Tm is thiamphenicol.

### 3.6 Whole-genome sequencing confirmed Ф027 deletion

**Figure 6:**
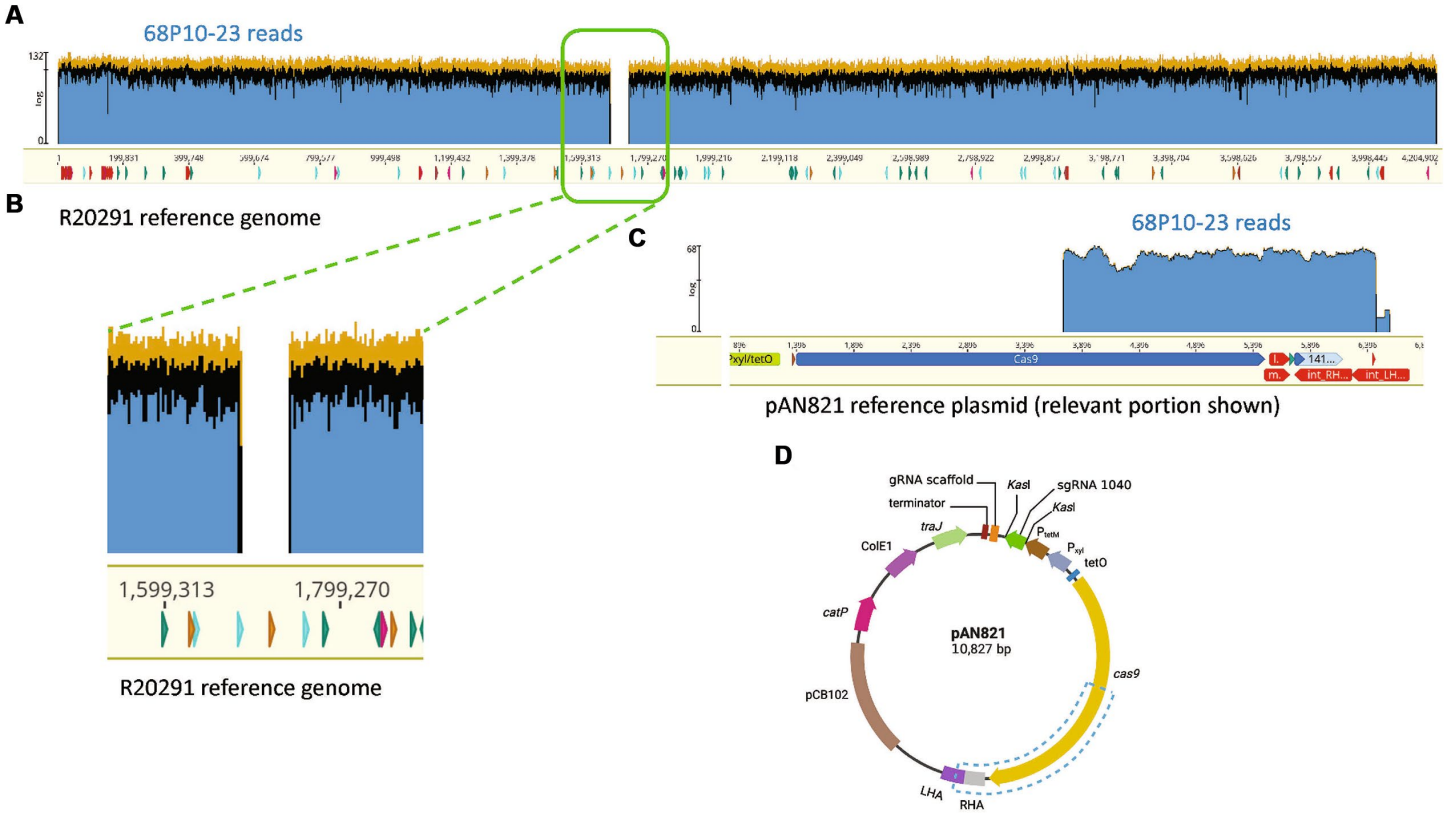
R20291Δϕ027 (68P10-23) contained a fragment of the mutagenesis plasmid. **(A)** Sequence reads of 68P10-23 (top blue bar) mapped to WT R20291 genome (bottom yellow bar with nucleotide numbers) showed missing reads as a gap, which belonged to the prophage, indicating its deletion. **(B)** Magnified section of where prophage sequence reads were missing from the WT genome. **(C)** A population of reads not mapping to R20291 was aligned to a 2.7 kb fragment of the pAN821 plasmid (bottom yellow bar) encompassing part of *cas9* and downstream sequences. **(D)** Blue dashed box shows the location of the 2.7 kb plasmid portion remaining in the prophage deletant. Created with biorender.com.

**Figure 7:**
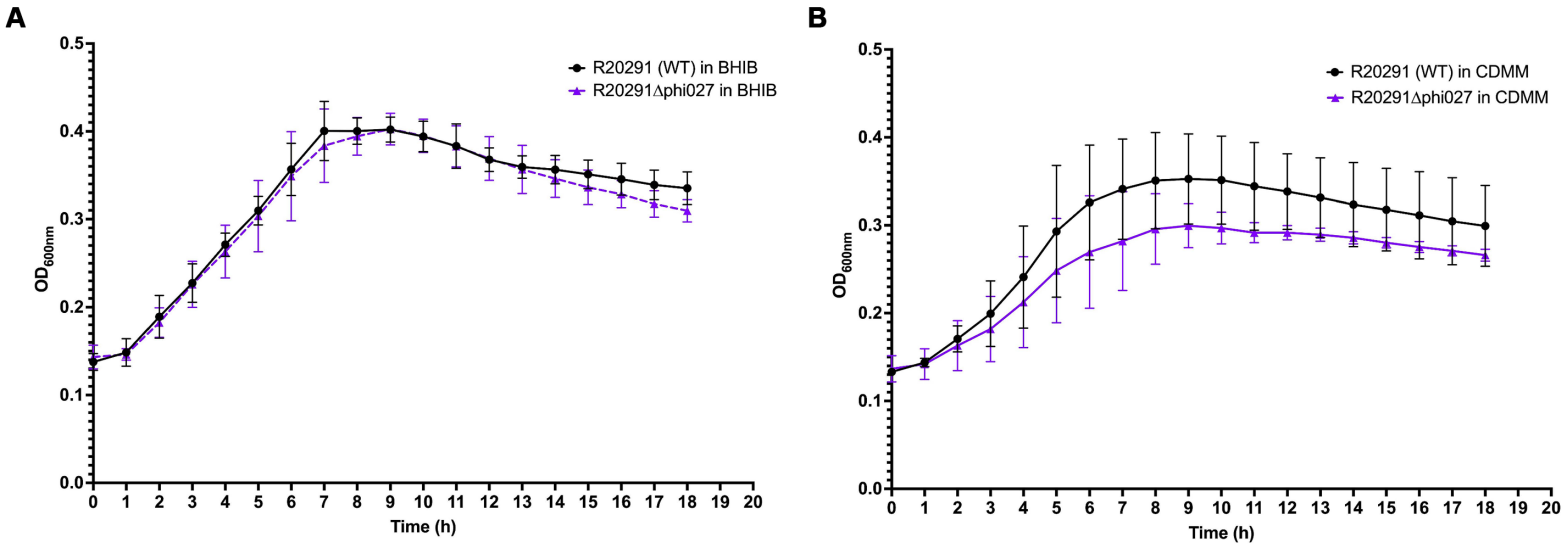
Growth of R20291 WT and Δϕ027 in **(A)** BHIB and **(B)** CDMM (*n* = 4).

Two Tm^S^ prophage deletants, 68P10-23 and 68P10-30, were subject to Illumina sequencing and had identical sequences, having the phage attachment site but neither the ϕ027 prophage genome nor the entire pAN821. Also ORF 1465 (678 bp), which was downstream of the *attR* site, hence predicted to be a bacterial gene (Figure 3), was deleted. Essentially a 56.8 kb locus containing the prophage and ORF 1465, was removed at the expected locations of nt. 1670843..1726837 (Figures 6A,B). However, 48–52% of the sequence reads contained a 2.7 kb remnant of pAN821 where the prophage was previously integrated (Figure 6C). The 2.7 kb remnant of pAN821 aligned with 1767 of 4,107 bases of the 3′ end of *cas9*, a 262 nt downstream intergenic region, 500 nt of the RHA, and 204 nt of the LHA (Figure 6D). Its presence was confirmed by PCR in all of 254 single colonies of 68P10-23 tested, and all of 160 single colonies of 68P10-30 tested (results not shown). To see if ORF 1465 could be absent from the WT genome naturally due to prophage excision, PCR and Sanger sequencing was carried out to show that deletion of ORF 1465 was likely a consequence of the mutagenic plasmid (Supplementary Figure S6). There was no other genomic difference between the prophage deletants and the WT. Batch culture growth curves of R20291Δϕ027 (68P10-23) was similar to WT in rich medium but marginally reduced compared with WT in minimal medium (Figure 7).

## 4 Discussion

Large MGEs such as prophages and ICE use integrase enzymes to facilitate their entry and exit from the host chromosome. There are two general families, i.e., the tyrosine and the serine recombinases. The former requires an accessory protein, Xis to excise the MGE while the serine recombinases can mediate both integration and excision (Stark, 2014). In this work, we showed that a prophage could be deleted from the *C. difficile* genome by targeting the integrase and that the ICE Tn*5397* can be cleanly removed by targeting the conjugation region. These observations show that it should be possible to specifically remove any of the large integrated MGEs from the *C. difficile* genome and determine their contribution to the organism’s biology. In this work, we ruled out a direct role for Tn*5397* in the transfer of the PaLoc. However, *C. difficile* does contain many different ICE and phages which have the potential to mediate chromosomal transfer, and systematic deletion of each of these is required to determine their precise role in the organism’s biology and their contribution to the wider mobilome.

Tn*5397* and the closely related genetic element Tn*916* are both very stable in bacterial genomes. The rate of loss of Tn*5397* being much less than the 2 in 600 observed in this work; we have tested 3,000 colonies containing wild-type Tn*5397* with no loss of the element (unpublished data). Therefore, it is likely that the deletion of part of the conjugation region destablises the element. There have only been a small number of studies examining gene regulation in the Tn*916/5397* family in genetic elements and these have all been done in Tn*916* (Scornec et al., 2017). This work has shown that transcription is tightly regulated and expression of the conjugation region requires transcription initiating at the strong *tet*(m) promoter progressing over the joint of the circular form into the conjugation region. Our mutant that lacks part of the conjugation region still contains the ends of the element on which TndX can act (explaining why circular forms of the element are still detected). It is possible that the deletion of part of the conjugation region results in premature transcriptional termination and that not enough TndX is produced to allow reintegration, hence the element is lost. However more work is required to determine the exact mechanism for loss of Tn*5397.*

Our hypothesis for transfer of the PaLoc is that integrated origins of transfers (*oriT*) result in the mobilization of the chromosome from donor to recipient (Brouwer et al., 2013). As Tn*5397* was the nearest *oriT* to the PaLoc this seemed like a good candidate for mobilizing the PaLoc. However, as the PaLoc still transferred from strains lacking Tn*5397,* this element is obviously not required for PaLoc transfer. The *C. difficile* genome does contain a number of integrated *oriTs* that could transfer the PaLoc, or it may transfer by a completely different mechanism, for example cell fusion to form a zygote. Further work is required to determine the exact mechanism of PaLoc transfer.

Clean deletion of large DNA fragments (up to 8,000 bp) in Clostridia using CRISPR-Cas9 is challenging (Wang et al., 2016, 2018). The potential causes include: (i) the Cas9 protein is toxic to the host; (ii) the Cas9-carrying plasmid is often large and therefore potentially unstable; (iii) homologous arms present on the mutagenesis plasmid enable repeated re-integration after double crossover events. Here, we demonstrate the successful deletion of the ~56 kb ϕ027 prophage in R20291 using the pMTL83151 backbone modified with CRISPR-Cas9. Four previous studies which described gene deletions in *C. difficile* 630 or R20291 by CRISPR-Cas 9 or Cas12a were built on pMTL84151 (McAllister et al., 2017), pMTL82151 (Hong et al., 2018; Wang et al., 2018), and pMTL83151 (Ingle et al., 2019). The main difference between these plasmids is the replicon, with pMTL82151 having a replicon from pBP1, pMTL83151 a replicon from pCB102, and pMTL84151 a replicon from pCD6 (Heap et al., 2009). Compared to other reports, our mutation efficiency of 0.3% for deleting Tn*5397* (21 kb) in *C. difficile* 630 was very low. This could be because of the size of the deletion and the limits of the Cas9 nuclease in *C. difficile* 630, since other studies which used Cas9 for selecting deletions at a high efficiency targeted sequences up to 3.6 kb. In *C. difficile* 630, Cas9 on pMTL82151 selected *spo0A* deletants (825 bp) at 100% efficiency (Wang et al., 2018), and Cas9 on pMTL83151 allowed selection of *pyrE* (234 bp), and *ermB1* and *ermB2* (3.6 kb) deletants at 89 and 96% efficiency, respectively (Ingle et al., 2019). Interestingly, Hong et al. was unable to obtain transconjugants when they attempted to use Cas9 on pMTL82151 for deletion of ϕCD630-2 (49 kb) in *C. difficile* 630. However, they succeeded using Cas12a (Cpf1) nuclease to select for deleted prophage ϕCD630-2 (49.2 kb) at mutation efficiencies of 37.5–58.3% (Hong et al., 2018). They also deleted *fur* (390 bp), *cwp66* (1.8 kb), *tetM* (1.9 kb), *ermB1* and *ermB2* (3.2 kb), and *tcdA* (8.1 kb) at mutation efficiencies of 25–100% (Hong et al., 2018). Our 50% mutation efficiency of deleting ϕ027 (56 kb) prior to plasmid curing in R20291 was comparable to McAllister *et al* (McAllister et al., 2017). They deleted *pyrE* (585 bp) and *selD* (951 bp) at 50 and 20%, respectively (McAllister et al., 2017). However, we were unable to completely cure the mutagenesis plasmid. It is worth noting that (Maikova et al., 2019) re-programmed the endogenous Cas I-B system in R20291 and achieved 90% mutation efficiency for deleting a 261 bp gene. This strategy could be useful to avoid toxic effects of Cas9.

In this work, the pAN821 mutagenesis plasmid deleted the ϕ027 prophage and a downstream predicted bacterial gene (CDR20291_1,465) from R20291. However, the plasmid was not completely cured; a truncated *cas9* and the RHA from the plasmid remained stably integrated in a population of bacterial cells. This likely occurred from an imprecise double crossover event and was detected by whole genome sequencing, although previous studies in *C. difficile* did not report this phenomenon, perhaps because it is undetectable by standard PCR assays for loss of gene targets. Primers flanking the *attL* and *attR* sites (LCF887/888, Figure 4) were not used to check for prophage deletion to avoid false positive results of prophage deletion, since the prophage could spontaneously exist extrachromosomally. A study in *C. beijerinckii* reported difficulties in plasmid curing, which was overcome with the inclusion of CRISPR-Cas9 self-targeting of the mutagenesis plasmid (Wang et al., 2016). This could be explored in future. It was not possible to quantify the subset of cells based on the number of sequence reads due to amplification bias in sequencing. However, cells with the plasmid remnant appear to be the dominant cell type based on PCR screening of single colonies. The truncated *cas9* translates to amino acid (aa) residues 781 to 1,368, which consists of the HNH, RuvCIII, Topo-homology, and PI domains that function in nuclease and PAM recognition, i. e. the “nuclease lobe” (Jinek et al., 2014; Nishimasu et al., 2014). However, without the other Cas9 protein domains that form the “recognition lobe” for facilitating guide RNA binding to DNA, this truncated *cas9* will likely be inactive if translated in the prophage deletant (Jinek et al., 2014; Nishimasu et al., 2014). Interestingly, Ingle et al. used the same backbone pMTL83151 for CRISPR-Cas9 deletion in *C. difficile* 630 and found truncation of Cas9 (Ingle et al., 2019). However, their truncated Cas9 was missing 87 aa from the N-terminus (Ingle et al., 2019) while the *cas9* remaining in our ϕ027 deletant would be missing approximately 780 aa (or 2,340 bases) from the N-terminus (or 5′ end of the gene) as mentioned above, if it was translated.

The *C. difficile* gene deleted adjacent to the prophage CDR20291_1465 is homologous to a putative manganese-containing catalase found in the *Bacillus subtilis* spore coat protein CotJC. In R20291, two other genes encode CotJC1 (CotCB) and CotJC2 (CotD), which have 70 and 50% amino acid sequence similarity, respectively, to CotJC (Permpoonpattana et al., 2011). Insertional mutations of *cotCB* and *cotD* in *C. difficile* 630 resulted in a reduction of catalase activity, but otherwise no significant defect to spore coat formation (Permpoonpattana et al., 2013). This suggests functional redundancy of CDR20291_1465 in R20291, the deletion of which would be unlikely to affect spore coat formation.

We were able to assay for ϕ027 plaque formation and hence propagate the phage using NCTC11207, although it was of a different ribotype to R20291 and contained two predicted prophage genomes. This indicates the potential of ϕ027 to lysogenize isolates other than RT 027, in which it is commonly found (He et al., 2013). Sensitivity of R20291 to ϕ027 was restored after prophage deletion, indicating in R20291 superinfection exclusion provided by the lysogenic prophage was the main mechanism of superinfection immunity. For instance, ϕ027 prophage encoded an Abi-like protein with similarity to *abiD* from *Lactococcus lactis*. Abi proteins protect uninfected bacterial cells from phage infection by infected cell suicide, hence aborting further phage infection (Lopatina et al., 2020). CRISPR-Cas is another system which provides immunity against phage. CRISPR arrays found in R20291 did not target ϕ027, though it is noteworthy that ϕ027 carried two CRISPR arrays which very likely conferred immunity to 12 phages (Boudry et al., 2015). Toxin-antitoxin systems, which is another possible phage defense system, has not been predicted in R20291. Since ϕ027 harbored two phage-defense systems, its deletion from R20291 may increase bacterial susceptibility to phage infection. The prophage deletant did not differ significantly in growth compared to WT in rich or minimal medium, although its growth in minimal medium was consistently lower than WT under nutrient limiting conditions (i.e., in the late stationary and death phase of growth curves) and could indicate the prophage was involved in regulation of genes for survival under those conditions. Our prophage deletion approach resulted in an unexpected genetic feature in the prophage deletant that could affect bacterial behavior. This could be determined by re-lysogenizing the prophage deletant with ϕ027 and comparing it to WT. Nevertheless, we anticipate the R20291 prophage deletant to be a useful strain for investigating prophage contribution to host virulence, fitness, and physiology, and a platform for other mutagenesis studies aimed at functional gene analysis without native phage interference.

In conclusion, we have shown that it is feasible to make a clean deletion of the ICE Tn*5397.* A phage genome could also be precisely deleted from the host chromosome. However, it was also observed that a fragment of the vector used for generating phage deletion could not be completely removed from the host cells. This is probably due to continual recombination between the host genome and the vector DNA. We have also observed this type of interaction with other host vector systems (Hussain et al. unpublished). Our previous work has also shown that some vectors can transfer between *E. coli* and *C. difficile* without the requirement for an obvious *oriT* and that transfer is sensitive to DNase (Khodadoost et al., 2017). Therefore, it is recommended that researchers undertake whole genome sequence analysis after mutant construction to determine the exact genotype of their mutant as this could impact how downstream physiological experiments are interpreted. Furthermore, it is important that further work is done to get a deeper understanding of the mechanism of transfer of MGEs between *C. difficile* strains.

## Data availability statement

The datasets generated for this study can be found in BioProject, accession number: PRJNA993731 and GenBank, accession number: CP129979.1. Materials generated in this study are available upon request from the corresponding author.

## Author contributions

HH: Data curation, Investigation, Methodology, Visualization, Writing – review & editing, Writing – original draft. AN: Writing – original draft, Writing – review & editing, Data curation, Investigation, Methodology, Visualization. CR: Methodology, Resources, Visualization, Writing – review & editing, Data curation, Formal analysis. KI: Formal Analysis, Methodology, Writing – review & editing. DK: Data curation, Resources, Writing – review & editing. VP: Investigation, Visualization, Writing – review & editing. PM: Conceptualization, Funding acquisition, Methodology, Project administration, Resources, Supervision, Validation, Writing – original draft, Writing – review & editing. SG: Conceptualization, Funding acquisition, Investigation, Methodology, Project administration, Resources, Supervision, Validation, Visualization, Writing – original draft, Writing – review & editing.

## Footnotes

^1^ https://www.benchling.com/

^2^ https://github.com/rrwick/Filtlong

^3^ https://pubmlst.org/
