# Supplementary material for: Removal of mobile genetic elements from the genome of Clostridioides difficile and the implications for the organism’s biology
Source: Front Microbiol. 2024 Jun 20;15:1416665. doi: 10.3389/fmicb.2024.1416665 (PMC11222575; doi:10.3389/fmicb.2024.1416665)
Supplement: Supplementary file 1 [file Data_Sheet_1.pdf]

Supplementary Material

Removal of mobile genetic elements from the genome of *Clostridioides difficile* and the implications for the organism's biology

Haitham Hussain, Amer Nubgan, César Rodríguez, Korakrit Imwattana, Daniel R. Knight, Valerija Parthala, Peter Mullany, Shan Goh*

*** Correspondence:** Corresponding Author: s.goh5@herts.ac.uk

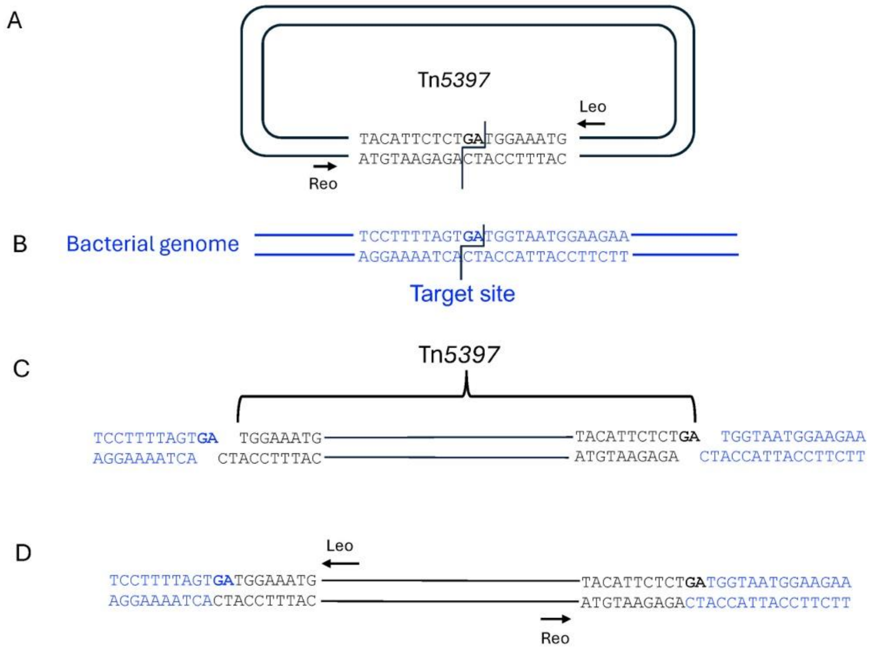

Supplementary Figure 1. Model for integration of Tn*5397*. A) Circular form of Tn*5397* (black). The DNA sequence at the joint of the circular form is shown. The GA cut site is underlined, and primer binding sites of Reo and Leo are indicated. **B)** The bacterial target sequence of the element is shown in blue, and the GA where TndX is proposed to cut is marked. **C)** Single strand overhangs at the ends of Tn*5397* and the target sites prior to integration of the element. **D)** An integrated form of Tn*5397* flanked by GA dinucleotides which are in bold. The location of the binding sites for the primers Reo and Leo are shown, and the direction of priming shown by the arrow.

A)

1	TTATATTAAA AAGTTTGGCA ATTAGGGTAA TTTTATAATA GATTAAATTT TAAACTTATG	
61	TAAGCTATAT GTAGTATTTA ATATTTTAAA AAGTATCTCA GAAAGATTTT TAATCAGAAT	
121	GAGGTGCTTT TTCATTTTTA TATAAAATAT TAAGTAATTT CAAAAAAATA TAAATTTATT	
181	TAATTTAATT AAAATATTAT AATATATTAG TTACATATTA AAATATATAA TCGAGATATT	
241	GTTAAGACAT TGTCGAGATA TTATAATATA TAACTAATGT ATTATAAATA ATAGTTGTAA	
301	ACTTCCTGTG ACATATATTT TAAATGCTTA TTCTTAAATT TTTGCATTAT TTATTAAAAT	
361	TTATTATGCT TTAACACTAA ATTGAGTTTG ATAATTATAA AACAAATAAT CAAAAATAGT	
421	GTAAAAATTA ATACTTTTTG TTAATATTAA TA**GATAAAGA TAATCTAAGG TGGTG**ATTAA	**F450 Primer**
481	ACTGACAATA GAAGAATACA AAAACAAATA CTTTACTGGA AATAAAATCG TACTTTCTCT	
541	AAATAGTTTT GATAATAAAG AACTATACTA TCATGAAACT GAAAATATAT TAAATTTAAT	
601	AAATGAAATA GATAAATTAA CATCAAGTAA GATATTTAAA ACAATAGAAG ATAAAAATGA	
661	TTTGGCACTA **GAGTCTTATT ATTCTAGTGT** AATAGAAGGT GCTTTTTCAA CTAGAAAGAT	**LHA start**
721	TGCAAAATCT ATAATTAGAG GAAAGATGAA ACCAAGTAAT AAAAGTGAAT ATATGATTTA	
781	CAATAATCAT AGAGCCTTAG AATATGGATT AGATAACTTA GATGAATTGT ATAGTCACAA	
841	ATTTATATAT GATTTGCATC ATATATTGGG CGAAAACTGC TTGGATTCAG AAGAATATGA	
901	GTATAGAACT GAAAAAGTAT ATGTATGTGA TTCAAAAGGT GAGATTATAC ATACAGGGCT	
961	TGAACCTTTG AAGATATATG ACTTTATGAG TAAATTAATA GATTTTATGG AAAATAGCAA	
1021	AGTAAGTAAT TTAATTAAAA GTGCTATAAT TCATTTTTAC TTTGTATATG TTCATCCTTT	
1081	TAGT**GA**TGGA AATGTACCAT CAAGACACCT GCTAAGAACC GCATAACAGC AGGCAAACAA	**GA site**
1141	CTTCATATCA AAATTTAAAA TTGAATAAAT ACATATAAAG AGCCATTTGA TTTTTCATAT	
1201	CAAGTGGTTT TT**GTTATGTA TATGGAGCAA GACGC**TTACT **AATTCCATTA TGGGAAATAG**	**LEO – LHA end**
1261	GAGCGTCTAT TTTTTTACCC AATTTTGAAA GGACGTGATA CCACGAAAGC AAAAAATCAA	
1321	GGTCGTTCCC CACCCAATAG ACCATCAAAC AAATTAGATT CATGAAACCA TTAAACAGAA	
1381	AGGATAGGTA AAAATATGGA ACTTAAATTT GTCATTCCCA ACATGGAAAA AACATTCGGA	

B)

```
21481  ACTGAACAAG CAGAGGTAGT GCAAAGCTAT AGAGAATTAA AGTCAAAAAT AAAATCCGAA
21541  GAGGAGAAAG TACAGGGTAC GGATAGATTC CTTGAAACTA TTAGTAAATA CAAAGATGTT
21601  ACAGAGTTAA ACCGTTCTAT GCTGTGTGAG CTGATTGATT CAATTTACGT GTATCAAGCA   REO
21661  GAGGGAATCG GTAAAGAACG TACTCAAAAA GTAGAAATCA ATTATAGGTT TCTAGCAGTG
21721  TCTCATTGAT ACATTCTCTGA TGGTAATGG AAGAACATCA AGAGCCTTAT CATATCTATA
21781  TTTGATTGAT AAAGGATATG ATACTTTCAA GGAGTTTTCA ATATCATATA TGATATCTAA
21841  AAACAGAACT AAGTATTATA AGGCTATACT TGATGTAGAA AACAAAGGAA ATAATTTAAC
21901  TGTATTTATA GAATTTATGC TCAAGTCTAT AATACAATCT ATTAATGAGA TGCGTAATAT
21961  GCATGATAGA AAATCTTTAG AAAGTATATT AAAAGAGGAA TTGTTTGAAA ATGACATAAC
22021  TCTATCAGCA ACAGAAGAAC ATATATTAAA ATATATTTGC AATAAAGATA ACTATTCTAT
22081  GACATTAGAA AATTATATAA AAAAGAATAA AAGTAGATAC TTAAAGGCTG GAATAAAGGA
22141  AATTGAATTA GTTGACCAGT TGATGGAAGT GTTTAATAAT CTTGAAGAGA TAGAGATTTT
22201  GTCTAAAGAA AAAAATATAT ATAAAGTTAA TGATAAGTAC TTAAAAATGC TTGATATAGA
22261  CTAATTTAGA TAATCTGGAG AGGGAATATA TGAATAATAA GAAAAAGGCA TTTTTGATTT   R22270 Primer
22321  CTGGGGCTAT AAACGTAATT TCATTAATTA TTGTTTTTTC ATTGTTTCAT TATAATATTA
22381  TAGAAAATAG TACACAAAAT ATGATAATAA CTGTAATCTT AATTTTTATT TTTGAAATGA
22441  TAAAAATAAA AATCATAGAG AAATACTATA AACTATAAAC AAGAAATATC TTTTCTAAGA
22501  GCATATTGTA CAATATGCTC TTAATTTAGT TATATAGATT TAATATAAAT AATGTATTTT
```

C)

```
1      TTATATTAAA AAGTTTGGCA ATTAGGGTAA TTTTATAATA GATTAAATTT TAAACTTATG
61     TAAGCTATAT GTAGTATTTA ATATTTTAAA AAGTATCTCA GAAAGATTTT TAATCAGAAT
121    GAGGTGCTTT TTCATTTTTA TATAAAATAT TAAGTAATTT CAAAAAAATA TAAATTTATT
181    TAATTTAATT AAAATATTAT AATATATTAG TTACATATTA AAATATATAA TCGAGATATT
241    GTTAAGACAT TGTCGAGATA TTATAATATA TAACTAATGT ATTATAAATA ATAGTTGTAA
301    ACTTCCTGTG ACATATATTT TAAATGCTTA TTCTTAAATT TTTGCATTAT TTATTAAAAT
361    TTATTATGCT TTAACACTAA ATTGAGTTTG ATAATTATAA AACAAATAAT CAAAAATAGT
421    GTAAAAATTA ATACTTTTTG TTAATATTAA TAGATAAAGA TAATCTAAGG TGGTGATTAA   F450 Primer
481    ACTGACAATA GAAGAATACA AAAACAAATA CTTTACTGGA AATAAAATCG TACTTTCTCT
541    AAATAGTTTT GATAATAAAG AACTATACTA TCATGAAACT GAAAATATAT TAAATTTAAT
601    AAATGAAATA GATAAATTAA CATCAAGTAA GATATTTAAA ACAATAGAAG ATAAAAATGA
661    TTTGGCACTA GAGTCTTATT ATTCTAGTGT AATAGAAGGT GCTTTTTCAA CTAGAAAGAT   LHA start
721    TGCAAAATCT ATAATTAGAG GAAAGATGAA ACCAAGTAAT AAAAGTGAAT ATATGATTTA
781    CAATAATCAT AGAGCCTTAG AATATGGATT AGATAACTTA GATGAATTGT ATAGTCACAA
841    ATTTATATAT GATTTGCATC ATATATTGGG CGAAAACTGC TTGGATTCAG AAGAATATGA
901    GTATAGAACT GAAAAAGTAT ATGTATGTGA TTCAAAAGGT GAGATTATAC ATACAGGGCT
961    TGAACCTTTG AAGATATATG ACTTTATGAG TAAATTAATA GATTTTATGG AAAATAGCAA
1021   AGTAAGTAAT TTAATTAAAA GTGCTATAAT TCATTTTTAC TTTGTATATG TTCATCCTTT
1081   TAGTGATGGA AATGTACCAT CAAGACACCT GCTAAGAACC GCATAACAGC AGGCAAACAA   GA site
1141   CTTCATATCA AAATTTAAAA TTGAATAAAT ACATATAAAG AGCCATTTGA TTTTTCATAT
1201   CAAGTGGTTT TTGTTATGTA TATGGAGCAA GACGCTTACT AATTCCATTA TGGGAAATAG   LEO - LHA end
1261   GAGCGTCTAT TTTTTTACCC AATTTTGAAA GGACGTGATA CCACGAAAGC AAAAAATCAA
1321   GGTCGTTCCC CACCCAATAG ACCATCAAAC AAATTAGATT CATGAAACCA TTAAACAGAA
1381   AGGATAGGTA AAAATATGGA ACTTAAATTT GTCATTCCCA ACATGGAAAA AACATTCGGA
```

D)

```
5341   CAATGAAGGA GAATTAGTCG GTGCTTGGTT TACCTTTCCC ATTGATTTTG AAGAAGTCAA
5401   AGAGAAAATC GGCTTGAATG ATGAATACGA GGAATACGCC ATTCACGATT ATGAGTTACC
5461   CTTTACGGTT GACGAATACA CTTCTATTGG CGAACTCAAT CGCCTATGGG AAATGGTATC
5521   GGAGTTGCCA GAGGAACTAC AATCCGAGCT ATCTGCTCTG CTCACTCATT TTTCAAGTAT   RHA1 beginning
5581   TGAGGAACTA AGCGAACATC AAGAGGATAT TATTATTCAC TCGGATTGTG ATGATATGGA   deletion end
5641   AGATGTTGCC CGTTACTACA TTGAAGAAAC TGGTGCTTTA GGCGAAGTAC CAGCCAGTCT
5701   TCAAAACTAT ATTGATTACG AATCCTATGG TCGTGATTTA GAACTTTCGG GAACATTCAT
5761   TTCTACCAAT CATGGGATTT TTGAAATCAC TCATTAAGTC TGTCGGTACA TTACTACTGG
5821   CAGAGTTTCT TGTTTGCGGG GTAGCTTAAA CAGCTATCCC TATTTTTATG AAAGGATTGA
5881   TTCTATGAAG AAAATACGAA GCTATACCAG TATTTGGTCT GTTGAGAAAG TGCTGTACTC
5941   CATAAACGAT TTTAGACTTC CGTTTCCCAT AACCTTTACG CAAATGACGT GGTTTGTCGT
6001   GTCACTGTTT GCGGTTATGA TACTTGGCAA CGTGCCACCT CTTTCCATGA TTGAGGGAGC
6061   ATTTCTCAAA TACTTTGGGA TTCCTGTCGC TTTCACATGG TTTATGTCTA CAAAAACCTT   RHA1 end
6121   TGATGGTAAA AAGCCTTATG GATTTTTAAA GTCTGTCATT GCTTATGCAC TGCGACCAAA
6181   GCTGACCTAT GCAGGGAAAA AAGTAACCCT TGGCAGAAAT CAGCCACAAG AAGCCATTAC
6241   AGCAGTTAGG AGTGAATTTT ATGGCATATC CAATTAAATA CATTGAAAAT AATCTGGTCT
6301   GGAATAAAGA TGGCGAATGT TACGCCTATT ATGAGCTTGT TCCTTACAAT TACTCATTTC   R6630 primer
6361   TAAGTCCAGA ACAGAAAATA CAAGTGCATG ATTCTTTCAG ACAGCTTATC GCACAAAATC
6421   GTGATGGCAA GATTCATGCC TTACAAATCA GTACAGAATC CAGCATACGT TCAGCACAAG
```

Supplementary Figure 2. DNA sequence of Tn*5397* and flanking regions. **A)** DNA sequence of the left end of Tn*5397* and flanking regions. The GA dinucleotide at the start of Tn*5397* is shown in red and the sequences inside the transposon are shaded in yellow. The extent of the LHA homology arm is marked (LHA start and LHA end) and shown as dark red text in the figure. The primer binding sites for F450 and Leo are also shown, marked in bold. **B)** DNA sequence of the right end of Tn*5397* and flanking regions. The GA dinucleotide at the end of Tn*5397* is shown in red and the sequences inside the transposon are shaded in yellow. The binding sites of primers Reo and R22270 are shown. **C)** DNA sequence of the left end of Tn*5397* showing the location of the 5 kb deletion of part of the conjugation region. The extent of the LHA homology arm is marked (LHA start and LHA end) and shown as dark red text in the figure. The primer binding sites for F450 and LEO are also shown, marked in bold (as in A). Part of the DNA sequence that has been deleted in *630*Δ*erm*::Δ*Conj* but is present in Tn*5397* has been shaded in yellow. **D)** Right end of the deletion point in *630*Δ*erm*::Δ*Conj*. The extent of the RHA homology arm is shown (RHA1 start and RHA1 end). The location of the primer binding site R6630 is shown in bold text. The end of the region that is deleted in *630*Δ*erm*::Δ*Conj*, but is present in Tn*5397*, is shaded in yellow.

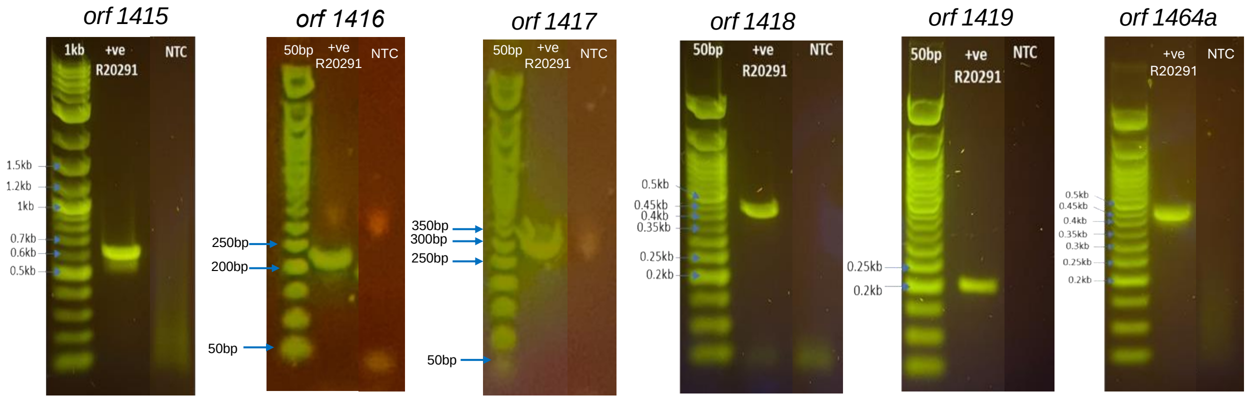

Supplementary Figure 3. PCR detection of predicted ϕ027 prophage sequences in R20291. Primers phiR2_1415_F/R, phiR2_1416_F/R, phiR2_1417_F/R, phiR2_1418_F/R, phiR2_1419_F/R and phiR2_1464a_F/R specific for prophage *orf 1415*, *1416*, *1417*, *1418*, *1419* and *1464a* were used to confirm prophage presence in genomic DNA of R20291. Expected amplicon sizes of 624 bp for *orf 1415*, 229 bp for *orf 1416*, 347 bp for *orf 1417*, 433 bp for *orf 1418*, 202 bp for *orf 1419*, and 445 bp for *orf 1464a* were obtained, and no template controls (NTC) were negative. DNA ladders used were either 1 kb Plus or 50 bp Plus DNA ladders from NEB, UK.

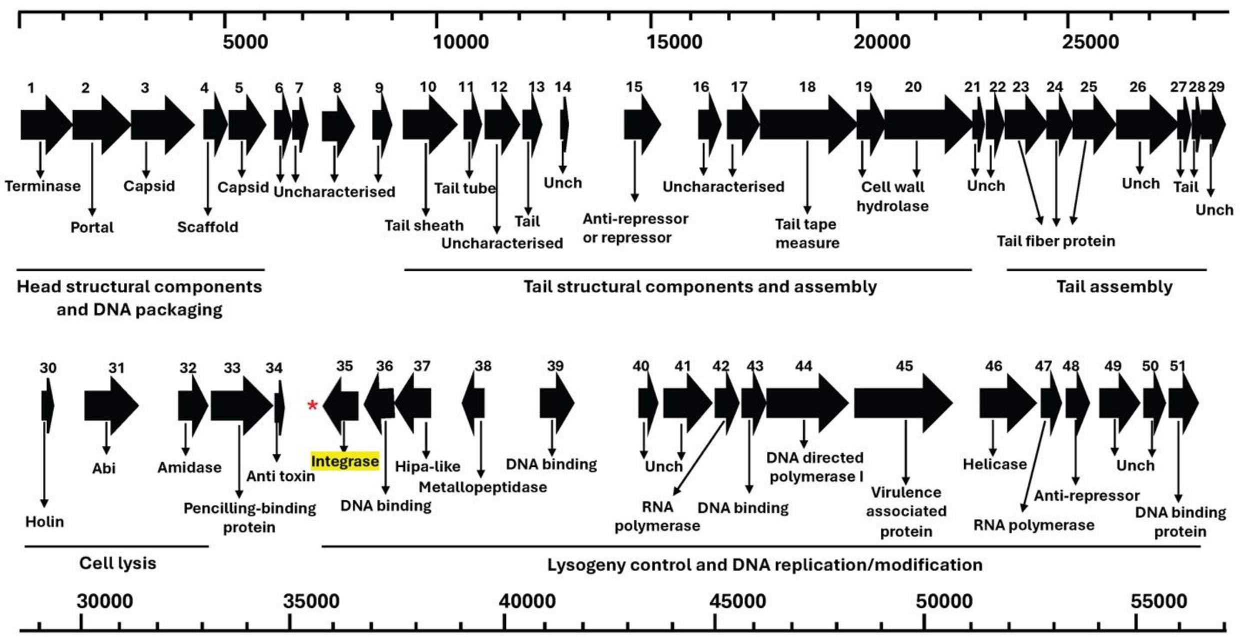

Supplementary Figure 4. Linear map of 56 kb ϕ027 phage genome. Direction of transcription and predicted ORFs (by convention of Genbank Accession number FN545816.1) are represented by arrows. The number of bases indicated in the ruler below and the ORF numbers assigned above relate to R20291 genome nucleotide positions and CDS annotations shown in Supplementary Table 4. Predicted gene functions and modular cassettes typical of phages are indicated. Location of *attP* is indicated by a red asterisk, in between a putative anti-toxin (ORF 1464a) and integrase (ORF 1415).

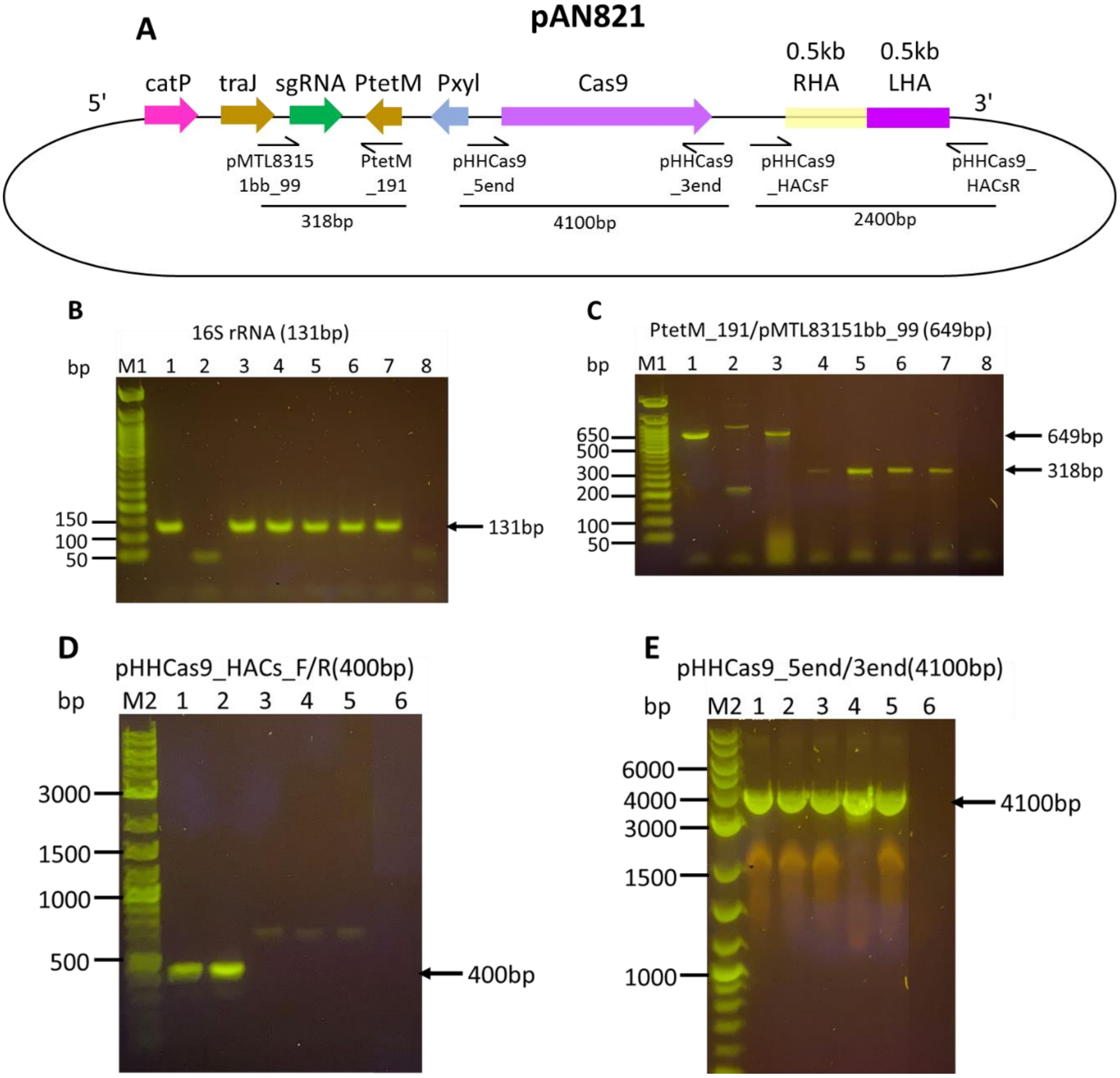

Supplementary Figure 5. Verification of selected plasmid features in *C. difficile* transconjugants. M1: 50bp Plus DNA ladder (NEB), M2: 1kb Plus DNA ladder (NEB). (**A**) Map of pAN821 showing PCR primer target sites. Colony PCR detection of (**B**) 16S rRNA and (**C**) sgRNA in the following samples: lane 1. pPM100 (positive control); 2. *E. coli* CA434 (negative control); 3. R20291 transconjugant of pPM100; 4. R20291 transconjugant of pAN721; 5-7. R20291 transconjugants of pAN821; 8. No template control. pPM100 contains *lacZ* instead of sgRNA, yielding a 649bp amplicon with pMTL83151bb_99/PtetM_191 primers. Non-specific amplicons were seen in CA424. PCR detection of (**D**) homology arm cassette and (**E**) *cas9* in the following R20291 gDNA samples: lane 1. Transconjugant of pPM100; 2. Transconjugant of pAN721; 3-5. Transconjugants of pAN821; 6. No template control. pPM100 and pAN721 lacking the homology arms cassette yielded a 400 bp amplicon in D, while faint bands at ~700 bp were non-specific and indicated that pAN821 integrated into the bacterial chromosome at either RHA or LHA, forming new host-plasmid junctions that did not contain target sizes for pHHCas9_HACSF/R primers.

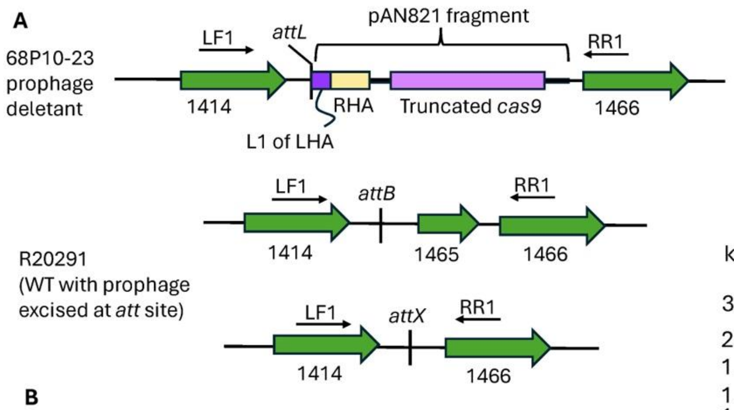

B

Strain	Feature presence			LF1/RR1 amplicon (kb)
	L1 & RHA	trcas9	1465	
68P10-23	+	+	-	3.3
WT	-	-	+	1.3
WT	-	-	-	0.5

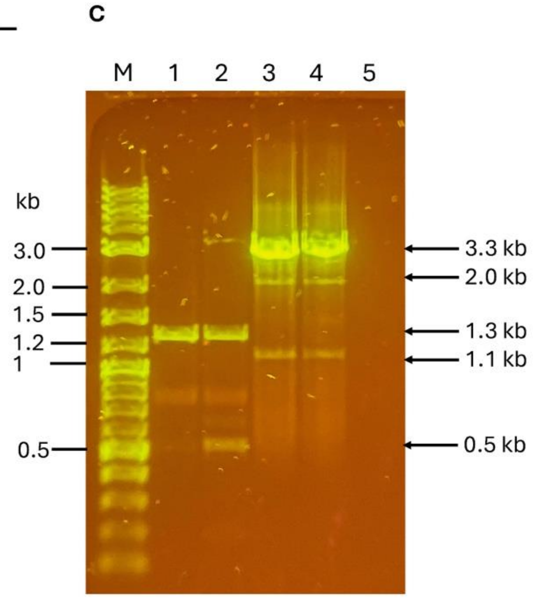

Supplementary Figure 6. Detection of ORF 1465 on bacterial genomes. (A) The prophage deletant 68P10-23, containing a fragment of the mutagenesis plasmid pAN821, was missing ORF 1465. To determine whether ORF 1465 can naturally be absent from the WT genome, primers LF1 and RR1 were used for PCR. Prophage in WT spontaneously excised at the *attB* site would leave behind ORF 1465. Hypothetically, prophage excision at an alternative unknown attachment site (*attX*), could result in loss of ORF 1465. **(B**) A summary of expected amplicon sizes depending on relevant genomic features. **(C)** PCR showing major amplicons as expected for two different genomic DNA preparations of WT (0.5 kb 1.3 kb) and 68P10-23 (3.3 kb), as well as minor bands of unexpected sizes (1.1 kb and 2 kb), which were non-specific by Sanger sequencing. Sanger sequencing of the 0.5 kb band from WT confirmed it was also a non-specific amplicon, hence WT genomic DNA lacking ORF 1465 was not found. WT genomic DNA with an integrated prophage would have an amplicon of ~57 kb, hence would not be amplified under cycling conditions used here for short amplicons. M: 1 kb Plus DNA ladder (NEB), Lanes 1- 2: WT DNA, Lanes 3-4: 68P10-23 DNA, Lane 5: No template control.

Supplementary Table 1. Oligonucleotides used in this study

Oligo name	Sequence 5' -> 3'	Purpose
Cas9-F	GATAGAGTATAATTAAAATAAGCATGGATAAGAAATACTCAATAGG	Amplification of fragment of *cas9* gene
Cas9-R	CAGTCGACTCAGTCACCTCCTAGCTGAC	
Pxyl/tetO-F	GTCCCGGGTTAAGACCCACTTTCAC	Amplification of the Pxyl tetO promoter.
Pxyl/tetO-R	CCTATTGAGTATTTCTTATCCATGCTTATTTTAATTATACTCTAC	
gRNA (S15600)	GCGCCTGATAAGAGTATCTGTCACTG	Sequence encoding guide RNA (see text for more details)
gRNA (AS15600)	GCGCCAGTGACAGATACTCTTATCAG	
gRNA (S2500)	GCGCCTAACCCCCCGTATCTAACAGG	
gRNA (AS2500)	GCGCCCTGTTAGATACGGGGGGTTAG	
Arm1-F	CAGCGCGCTAAGGTGGTGATTAAACTGAC	Amplification of LHA of Tn*5397*
Arm1-R	CATAGAACGGTTTAACTCTGGCTGTTATGCGGTTCTTAGC	
Arm2-F	GCTAAGAACCGCATAACAGCCAGAGTTAAACCGTTCTATG	Amplification of RHA of Tn*5397*
Arm2-R	CTGCGCGCACTTCCATCAACTGGTCAAC	
Arm1-F1	CAGCGCGCACTAGAGTCTTATTATTC	Amplification of RHA2 (see Fig. 2)
Arm1-R1	CTCGGATTGTAGTTCCTCTGGCTATTTCCCATAATGGAATTAG	
Tn*5397*(F450)	AGATAAAGATAATCTAAGGTGGTG	Amplification of part of Tn*5397* and flanking sequence, binding sites are shown in **Fig. 2**
Tn*5397*(R6630)	GAACAAGCTCATAATAGGCGTAAC	
Tn*5397*(R22270)	CATATATTCCCTCTCCAGATTATC	
Tn*5397* (Leo)	GCGTCTTGCTCCATATACATAAC	Amplification of the Tn5397 circular form.
Tn*5397* (Reo)	CCGTTCTATGCTGTGTGAGCTG	
f027_gRNA1040_top	GCGCCAAAAAAGTAGAAAGAGTTGGG	Self-annealing primer pair to clone sgRNA1040 of 20 bases into KasI linearised pPM100 to target ϕ027 1415 gene. sgRNA 1040 targets the coding strand at nt 76..95 of 1415, with a PAM of tgg. Underlined bases indicate ends complementary to KasI sites.
f027_gRNA1040_bot	GCGCCCCAACTCTTTCTACTTTTTTG	

pHHCas9_HACS_F	TAATCGCCTTGCAGCACATC	To check cloning of ϕ027 1415 HA into *Bss*HII site of pPM100 and for Sanger sequencing
pHHCas9_HACS_R	GGCTTGATGTGTTGGTAGCA	
14152HA_pwalk1	TTGATGAAGCTATTAAAGCAGG	Sanger sequencing of HA2 cloned in pAN921
14152HA_pwalk2	TTCTACTATTAATAAATGCCAGC	
int_RLA_fwd	CAGGCTTCTTATTTTTATGGATGATACATATGAACTTTTAAAAAGAAGTG	Amplify 0.5kb right homolog arm (RHA) of ϕ027 *orf 1415* to clone with pAN721 linearised with *Bss*HII
phi027_1415_RHA_F	gtggcaaactAATAGTATCCCTCCCTTTTAGAATG	
int_RLA_rev	GTTCAAAAAAATAATGGCGGCTAATCTCCCCACTTTCTTACC	Amplify 0.5 kb left homolog arm (LHA) of ϕ027 *orf 1415* to clone with pAN721 linearised with *Bss*HII
phi027_1415_LHA_R	ggatactattAGTTTGCCACCCAAAAATATAATAC	
NF1643	GTGAGCCAGTACAGGATGGA	Detection of *C. difficile* 16S rRNA gene (1).
NF1644	AGGAGTTTGGACCGTGTCTC	
pMTL83151bb_99	CGACTCGGTGCCACTTTT	Checking insertion of gRNA into *Kas*I site of pPM100
PtetM_191	AAATATGCGGCAAGGTATTCTT	
pHHCas9_5end	CCTGGACTTCATGAAAAACTAAAAA	Detection of *cas9* in *C. difficile* transconjugants.
pHHCas9_3end	TCTCCATGGACGCGTGAC	
pHHCas9_3F	AACGGTCGTAAACGGATGCT	Detection of *cas9* 3' end junction cloned into pPM100
pHHCas9_3R	TCTGTCACTTTGCTCACCCC	
pHHCas9_3F	AACGGTCGTAAACGGATGCT	Detection of truncated *cas9* of pAN821 remnant integrated into chromosome of Tm^S prophage deletants.
pHHCas9_3end	TCTCCATGGACGCGTGAC	
catP_3	ATGGTATTTGAAAAAATTGATAAAAATAG	Detection of *catP* in transconjugants
catP_2	TTAACTATTTATCAATTCCTGCAATTCG	
LCF 890	GAAGCTACCTAGAAGGAAGGTATTT	Amplification of ϕ027 *attPP'* (i.e. phage genome that includes ORF1464 & 1464a), as predicted in (2).
LCF 889	AGACATGCACATGCTACTCTATT	
LCF 887	CCTATTGTAGCACCTAATGATGCATTGGAA	Amplification of ϕ027 *attBB'* (i.e. non-lysogenic bacterial chromosome excluding 1464 & 1464a), as predicted in (2)
LCF 888	CACCCAACTGTTCTTGCATTAAA	
LCF 890	GAAGCTACCTAGAAGGAAGGTATTT	Amplification of ϕ027 *attPB* (*attR*) right end (2)
LCF 888	CACCCAACTGTTCTTGCATTAAA	

LCF 887	CCTATTGTAGCACCTAATGATGCATTGGAA	Amplification of ϕ027 *attBP* (*attL*) left end (2)
LCF 889	AGACATGCACATGCTACTCTATT	
phiR2_1415_F	GCAGTCCATCCTTACAAACACA	confirm presence of *orf 1415* in ϕ027 gDNA
phiR2_1415_R	AGTTGATAATTGGCTATGACCCA	
phiR2_1416_F	GCCCATTTGCAAGTAGATTACCT	confirm presence of *orf 1416* in ϕ027 gDNA
phiR2_1416_R	TGTCCTTCACACCCAGCATA	
phiR2_1417_F	GAAACTGGAGAATATTGGGCAGA	confirm presence of *orf 1417* in ϕ027 gDNA
phiR2_1417_R	AGAGCCCAATTATTTTGATGCCT	
phiR2_1418_F	AGACGCACTTTTAGACTTAGCA	confirm presence of *orf 1418* in ϕ027 gDNA
phiR2_1418_R	TCCTAAGTCCAACATGCTTTTCT	
phi027_1419_F	TGGAGAATTTTGGTTTGTTGG	confirm presence of *orf 1419* in ϕ027 gDNA
phi027_1419_R	TTTTGCACTTGGCAACTTAGAA	
phi027_1464a_F	TCAATCTGGGTGTAAAAGAGCC	confirm presence of *orf 1464a* in ϕ027 gDNA
phi027_1464a_R	GGAGGTAAGAAAGTGGGGAGA	
phi027_1415_F1	CATACATTCTAAAAGGGAGGGATAC	To check sequence of *orf 1415* and verify sgRNA target sequence by Sanger sequencing
phi027_1415_R1	CCCATTTGCCACCGTATTAT	
M13F	TGTAAAACGACGGCCAG	Sanger sequencing primers
M13R	CAGGAAACAGCTATGACC	
68P1023_RF	ATTTTCCAATCAAAGATACTCTCA	Detection of pAN821 remnant integrated into chromosome of Tm^S prophage deletants
68P1023_RR	GATAAAGTTCTTAGTGCATATAACA	
68P1023_LF	CAATTGAGTATTTTCAACAGGA	Detection of pAN821 remnant integrated into chromosome of Tm^S prophage deletants.
68P1023_LR	AGCCTCATCATTCCAACTATACCAG	
LF1	GGAATGGTAAGACAATGGCAGA	Detection of ORF 1465 on bacterial genome after prophage excision.
RR1	ACTGTCCAAAATACCACCTCAT	

Supplementary Table 2. Guide RNA features

Strain	gRNA name	gRNA sequence (5' -> 3')	PAM	On-target score	Off-target score
630	2500-B	TAACCCCCCTATCTAACAG	GGG	79.2	88.9
630	15600-C	TGATAAGAGTATTGTCACT	GGG	63.8	53.3
R20291	1040	AAAAAAGTAGAAAGAGTTGG	TGG	98.4	32.3

Supplementary Table 3. Predicted features of the prophages identified in *C. difficile* NCTC11207

Prophage	Length (kb)	Total proteins	Chromosomal position	Most similar to phage[1]	GC (%)	Similarity to phi027[2]	Integrase type	Integration site
1	52.3	104	265759-318096	phiCDMH1_NC_024144 (33)	28.8	33.589	Tyrosine (with a predicted excisionase in the genome)	GTGATACTAAAATGATACTAT and ATTAGAAAAAAA
2	61	108	1345187-1406263	phiMMP01_NC_028883 (26)	28.5	38.136	Tyrosine, and serine	AGATAAAAAAAAT

[1]Number in parenthesis refers to the number of similar proteins between the prophage and phage indicated.

[2]Similarity calculated by pairwise nucleotide comparison of the indicated prophage to ϕ027 by VIRIDIC.

Supplementary Table 4. CDS of ϕ027 and their predicted function

ϕ027 ORF no. in Supplementary Figure 1	R20291 CDS*	FASTA Predicted function
1	CDR20291_1432	Terminase large subunit
2	CDR20291_1433	Phage portal protein
3	CDR20291_1434	Capsid
4	CDR20291_1435	Scaffold protein
5	CDR20291_1436	Putative phage major capsid protein
6	CDR20291_1437	Uncharacterized
7	CDR20291_1438	Uncharacterized
8	CDR20291_1439	Uncharacterized
9	CDR20291_1440	Uncharacterized
10	CDR20291_1441	Tail sheath
11	CDR20291_1442	Tail tube
12	CDR20291_1443	Uncharacterized
13	CDR20291_1444	Tail/portal
14	CDR20291_1445	Uncharacterized
15	CDR20291_1446	Antirepressor or repressor
16	CDR20291_1447	Uncharacterized protein
17	CDR20291_1448	Uncharacterized protein
18	CDR20291_1449	Tail tape measure protein
19	CDR20291_1450	Cell wall hydrolase
20	CDR20291_1451	putative phage cell wall hydrolase
21	CDR20291_1452	Uncharacterized protein
22	CDR20291_1453	Uncharacterized protein
23	CDR20291_1454	Phage tail protein
24	CDR20291_1455	Tail
25	CDR20291_1456	Phage tail fiber protein
26	CDR20291_1457	Uncharacterized
27	CDR20291_1458	Tail
28	CDR20291_1459	Tail
29	CDR20291_1460	Uncharacterized protein
30	CDR20291_1461	Holin
31	CDR20291_1462	Abi
32	CDR20291_1463	N-acetylmuramoyl-l-alanine amidase
33	CDR20291_1464	Penicillin-binding protein
34	CDR20291_1464a	Putative antitoxin component PemI
35	CDR20291_1415	Integrase

36	CDR20291_1416	DNA binding
37	CDR20291_1417	Hipa-like
38	CDR20291_1418	Metallopeptidase
39	CDR20291_1419	DNA binding
40	CDR20291_1420	Putative uncharacterized protein
41	CDR20291_1421	Putative uncharacterized protein
42	CDR20291_1422	RNA polymerase
43	CDR20291_1423	DNA binding
44	CDR20291_1424	DNA-directed DNA polymerase I
45	CDR20291_1425	Putative virulence-associated protein
46	CDR20291_1426	Helicase
47	CDR20291_1427	RNA polymerase
48	CDR20291_1428	Anti-repressor
49	CDR20291_1429	Uncharacterized
50	CDR20291_1430	Uncharacterized
51	CDR20291_1431	DNA-binding protein

* By convention of Genbank accession number FN545816.1.

Supplementary Table 5. Conjugation frequencies of *C. difficile* strain R20291.

<table>
<tr><th>Plasmids</th><th>Donor cell count (cfu/mL)</th><th>Recipient cell count (cfu/mL)</th><th>Transconjugant cell count (cfu/mL)</th><th>Conjugation frequency/ donor</th><th>Conjugation frequency/ recipient</th><th>Average frequency/ donor</th><th>Average frequency/ recipient</th></tr>
<tr><td rowspan="2">pPM100</td><td>1.6×10^{7}</td><td>3.2×10^{5}</td><td>4</td><td>2.5×10^{-7}</td><td>1.2×10^{-5}</td><td rowspan="2">1.31×10^{-7}</td><td rowspan="2">6.09×10^{-6}</td></tr>
<tr><td>1.8×10^{8}</td><td>1.2×10^{7}</td><td>2</td><td>1.1×10^{-8}</td><td>1.7×10^{-7}</td></tr>
<tr><td rowspan="2">pAN721</td><td>2×10^{7}</td><td>3.2×10^{5}</td><td>6</td><td>3×10^{-7}</td><td>1.9×10^{-5}</td><td rowspan="2">1.62×10^{-7}</td><td rowspan="2">9.71×10^{-6}</td></tr>
<tr><td>2.1×10^{8}</td><td>1.2×10^{7}</td><td>5</td><td>2.4×10^{-8}</td><td>4.2×10^{-7}</td></tr>
<tr><td rowspan="2">pAN821</td><td>2.3×10^{7}</td><td>3.2×10^{5}</td><td>6</td><td>2.6×10^{-7}</td><td>1.9×10^{-5}</td><td rowspan="2">1.4×10^{-7}</td><td rowspan="2">9.67×10^{-6}</td></tr>
<tr><td>2×10^{8}</td><td>1.2×10^{7}</td><td>4</td><td>2×10^{-8}</td><td>3.3×10^{-7}</td></tr>
</table>

References

1. Fagan RP, Fairweather NF. *Clostridium difficile* has two parallel and essential Sec secretion systems. J Biol Chem. 2011 Aug 5;286(31):27483-93.
2. Sekulovic O, Fortier LC. Global Transcriptional Response of *Clostridium difficile* Carrying the phiCD38-2 Prophage. Applied and Environmental Microbiology. 2015;81(4):1364-74.
